# Environmental and Genetic Variation for Yield-Related Traits of Durum Wheat as Affected by Development

**DOI:** 10.3389/fpls.2018.00008

**Published:** 2018-01-18

**Authors:** Francesco Giunta, Pasquale De Vita, Anna M. Mastrangelo, Gavino Sanna, Rosella Motzo

**Affiliations:** ^1^Sez. Agronomia, Coltivazioni erbacee e Genetica, Dipartimento di Agraria, University of Sassari, Sassari, Italy; ^2^Consiglio per la Ricerca in Agricoltura e L'analisi Dell'economia Agraria-Centro Cerealicoltura e Colture Industriali (CREA-CI), Foggia, Italy; ^3^Consiglio per la Ricerca in Agricoltura e L'analisi Dell'economia Agraria-Centro Cerealicoltura e Colture Industriali (CREA-CI), Bergamo, Italy

**Keywords:** phenology, kernel number, tillering, spike fertility, durum wheat, QTL, candidate genes

## Abstract

Phenology has a profound effect on adaptation and productivity of crops. The impact of phenology on tillering and fertility traits of durum wheat (*Triticum turgidum* L. subsp. *durum* Desf.) was evaluated with the aim of specifying which group of flowering genes (*Vrn, Ppd*, or *eps*) was involved in their control. A recombinant inbred line population was grown under four contrasting conditions of vernalization and daylength. Phenotyping was carried out according to robust phenological models dissecting both phenological and yield related traits. Whole-genome profiling was performed using the DArT-Seq technology. The genetic variability for tillering was mainly related to the genetic variability for vernalization sensitivity, as shown by the many quantitative trait loci (QTLs) identified in non-vernalized plants associated to both tillering and phenological traits. No effects of photoperiod sensitivity on spikelet number were detected in short-day-grown plants, apparently because of limited genetic variability in photoperiod sensitivity of the population. Earliness *per se* was involved in control of spikelet number via final leaf number, with these traits genetically correlated and sharing some QTLs. Chaff weight and number of kernels per g chaff were negatively associated and related to anthesis date under most conditions. QTL mapping uncovered novel loci involved in phenological control of tillering and fertility traits, and confirmed the presence of several well-established loci. Phenotyping of both phenology and kernel number according to a robust physiological model amplified the possibility of identifying genetic factors underlying their variations. Also, isolating known flowering gene cues by manipulation of environmental conditions provided the opportunity for each group of genes to be expressed without confounding effects of the others. This information helps to predict the consequences of either genetic manipulation of flowering genes and changes in environmental conditions on the potential yield of durum wheat.

## Introduction

Grain yield improvements in wheat have been highly associated with increased kernel number per unit area, with little change achieved for the individual kernel weight (Giunta et al., 2007; Foulkes et al., 2011, and references therein). The numerical yield component of kernel number per unit area can be further split into the number of fertile spikes per unit area and the number of kernels per spike, or the spike fertility. Both the number of tillers produced by a wheat plant, which is a component of the number of spikes per unit area, and the spike fertility are determined in part by the wheat development (Hay and Porter, 2006). Wheat development is a complex process that is controlled by several genes that are classified according to whether they respond to vernalization (*Vrn*) or the photoperiod (*Ppd*), or whether they confer earliness *per se* (*Eps*) (Snape et al., 1996, 2001; Worland, 1996; Law and Worland, 1997; Laurie et al., 2004). These genes modulate flowering time through their control of variations in the final leaf number, the phyllochron, and the duration of the flag leaf–anthesis interval (Sanna et al., 2014).

The emergence of the first leaf tiller coincides with the appearance of the tip of leaf 4. The subsequent primary tillers emerge at regular intervals of one phyllochron (Baker and Gallagher, 1983; Kirby et al., 1985; Masle, 1985). Around the time that the main shoot apex reaches the terminal spikelet stage, the tillers begin to die in the reverse order of their emergence (Hay and Kirby, 1991; Evers et al., 2006, and references therein). In bread and durum wheat, and in barley, the leaf number of the main shoot at which tillering ceases is correlated with the total number of leaves on the main shoot (Kirby et al., 1985; Giunta et al., 2015). The phyllochron and the final leaf number therefore set the upper limit to the number of tillers per plant (Kirby et al., 1985; Borràs et al., 2009). A number of quantitative trait loci (QTLs) that control tillering have been described in wheat, and generally in bread wheat (Kato et al., 2000; Li et al., 2002, 2010; Huang et al., 2003, 2004; Kumar et al., 2007; Naruoka et al., 2011; Jia et al., 2013; Yang et al., 2013; Zhang et al., 2013; Deng et al., 2015; Xie et al., 2016). However, in only a few cases have any attempts been made to relate these QTLs to flowering genes.

The spike-fertility trait that is more closely related to development is the number of spikelets per spike. The period of spikelet initiation on the apex begins when the interplay of the responses of the genotype to the photoperiod and to vernalization determines the switch from the vegetative to the reproductive phase (McMaster, 1997). Therefore, the timing of the transition of the main stem apex to the reproductive stage influences both the tillering ability and the spike fertility. The duration of spikelet initiation from the formation of the first reproductive primordium, or collar, to the appearance of the terminal spikelet, lasts at least 2–3 weeks (i.e., several hundred degree-days; Hay and Kirby, 1991). This is directly controlled by the temperature and the photoperiod (Kirby, 1990).

Spike fertility can also be analyzed in terms of the chaff weight and the number of kernels per g chaff (Stapper and Fischer, 1990; Motzo et al., 2011; Guo et al., 2017), and the developmental rate is also involved here. Variation in chaff weight is associated with phasic development via its association with both the spikelet number (Gaju et al., 2009) and the length of the stem elongation phase, when spike growth takes place. This latter period is crucial for spike fertility (Gonzáles et al., 2005; Slafer et al., 2009), and hence it is also linked to the number of kernels per g chaff.

To date, a number of QTLs have been found in wheat in terms of fertility traits, and these numbers have grown rapidly in the last few years (Miura et al., 1992, 1994; Araki et al., 1999; Huang et al., 2004; Zhou et al., 2006; Li et al., 2007; Cui et al., 2012; Hu et al., 2015), although only in a few cases has any relationship been established between fertility traits and development (Kato et al., 2000; Hu et al., 2015).

In the light of the tight link between both tillering and spikelet number and development, any breeding studies that are aimed at modification of these traits has to take into account their genetic and physiological interrelationships with the flowering genes. This might also include the need to discriminate the different impacts of these three groups of genes that control development.

The objectives of the present study were essentially two-fold: (i) to determine to what degree the variability in tillering and spike fertility traits is associated with the variability in development, and more specifically, whether the genotypic relationships between the tillering and spike fertility traits are differentially affected by *Eps*, or show differential sensitivity to the photoperiod or vernalization; and (ii) to identify the chromosomal locations of the genes and/or QTLs that influence the expression of tillering and spike-related traits under different environmental conditions.

## Materials and methods

### Plant materials and experimental design

This study was carried out at Ottava, Sardinia, Italy (41°N, 8°E, 80 m a.s.l.) across the 2010/11 and 2012/13 seasons with a RIL population bred from the cross between cv. “Ofanto” (an early flowering, semi-dwarf cultivar that was released in 1990) and cv. “Senatore Cappelli” (a late flowering, tall cultivar that was released in 1915). For the 2010–11 season, 98 RILs and the parents were used to compare three conditions that were previously described in Sanna et al. (2014): May-sown plants (thus with long daylight period) that were artificially vernalised (LD-V) and not vernalised (LD-NV) were grown in pots outdoors, and December-sown plants (with short daylight period) that were artificially vernalised (SD-V) were grown in pots in a greenhouse (Table 1). Two pots (each containing three plants) arranged in a completely randomized design were assigned to each RIL/treatment combination. Then this same RIL population, which included the parents, was sown in the field on 11 December 2012 (SD-FI). These seeds were not artificially vernalized, but their December sowing guaranteed some natural vernalization, in contrast to the LD-NV condition for the May sowing, which excluded any natural vernalization. The plots comprised six 1.2-m rows, each separated by 15 cm, laid out as a randomized complete block design with three replications.

**Table 1:** Conditions applied to the plants and prevailing thermal and photoperiod conditions.

**Treatment code**	**Number of RILs^a^**	**Description**	**Artificial Vernalization**	**Imbibition date**	**Transplanting/ sowing date**	**Daylength at transplanting (h)**	**Mean temperature (°C)**		
							**Transpl-LN_MAXTILL_**	**Transpl-ant**	**Ant-mat**
LD-V	100	Pots, outdoor	Yes	15 April 2010	24 May 2010	14.8	20.2	20.4	24.2
LD-NV	100	Pots, outdoor	No	21 May 2010	24 May 2010	14.8	20.0	21.6	22.6
SD-V	100	Pots, greenhouse	Yes	12 Nov 2010	23 Dec 2010	9.4	15.4	16.6	21.0
SD-FI	100	Field	No	–	11 Dec 2012	9.6	9.3	11.8	17.2
SD-V-IN	8	Pots, greenhouse	Yes	8 Nov 2012	17 Dec 2012	9.5	18.0	18.4	21.6
SD-NV-IN	8	Pots, greenhouse	No	10 Dec 2012	17 Dec 2012	9.5	18.0	18.5	21.8
SD-V-OUT	8	Pots, outdoor	Yes	8 Nov 2012	17 Dec 2012	9.5	9.3	11.7	17.3
SD-NV-OUT	8	Pots, outdoor	No	10 Dec 2012	17 Dec 2012	9.5	9.5	11.7	17.3

a *Parents included*.

In the 2012–13 season, another study was carried out in pots with a sub-set of six of these RILs and the two parents. The six RILs selected were those with the more extreme final leaf number (FLN) that was recorded for each of the three 2010–11 conditions. A set of similarly sized artificially vernalized and non-vernalized seedlings was transplanted into a total of 48 pots, both within a greenhouse (i.e., SD-V-IN, SD-NV-IN) and outdoors (i.e., SD-V-OUT, SD-NV-OUT) on 17 December 2012. Vernalization was achieved according to Sanna et al. (2014). Three pots (each containing three plants) were assigned to each condition × RIL combination. The pots were arranged according to a completely randomized design, and their relative positions were periodically changed. All of the experiments plants were grown under conditions of optimal water and nutrient availability.

### Phenotyping

In all of the experiments, the plants were monitored on a twice-weekly basis, to record the time of anthesis (ANT) of the main stem (i.e., stage 65; Zadoks et al., 1974). For the 2010/11 part of the study, the time of arrival at the “terminal spikelet” stage (TS) was estimated non-destructively from the measurements of the rate of elongation of the plantlets, as described by Sanna et al. (2014). For all of the study, the Haun stage (Haun, 1973) was recorded twice weekly, and used to calculate the regression against the accumulated thermal time, to obtain the rate of leaf appearance and its inverse, the phyllochron (PHY). These Haun stage measurements also allowed determination of time of the flag leaf appearance (FLA) and final leaf number (FLN).

Tillering was monitored throughout the study, except for the SD-FI group. The number of tillers per plant was recorded weekly during the Haun stage determination, and continued until no more tillers were produced for three subsequent sampling dates. At maturity, the number of tillers bearing spikes for each plant was counted, together with the number of fertile spikes. The tillering data were used to calculate the maximum number of tillers (MAXTILL), the number of fertile tillers (i.e., tillers bearing a fertile ear; FERTTILL), the percentage of fertile tillers (FERTTILL%), the Haun stage when MAXTILL was reached (LN_MAXTILL_), and the rate of tillering (RATE = MAXTILL/LN_MAXTILL_; number of tillers per emerged leaf).

At maturity the spikes of the main stems were harvested separately from the spikes on the tillers. The following measurements were determined separately on the main stem and tiller spikes: number of total spikelets (SPKLT); number of kernels per spike (K/SPIKE) and per fertile spikelet (K/SPKLT); and chaff weight (CHAFF). The number of kernels per g chaff (K/CHAFF) was obtained by dividing K/SPIKE by CHAFF (Gonzalez et al., 2011; Guo et al., 2017).

### Statistical treatment of the phenotypic data

For the whole RIL population, the statistical analysis was the same as that described in Sanna et al. (2014). For the subset dataset that included six RILs and the parents (i.e., the 2012–13 pot experiment), analysis of variance was performed to define any effects of vernalization and or the thermal regime (i.e., outdoor vs. greenhouse) on the traits measured according to a complete randomized design.

### Map construction and QTL mapping

An updated version of the “Ofanto” × “Cappelli” genetic map that was reported previously (Marone et al., 2012) was developed and used for the QTL analysis. Whole-genome profiling was performed using the DArT-Seq technology (Diversity Arrays Technology Pty Ltd, Australia). Briefly, the genetic map was composed of 32 linkage groups that covered all of the chromosomes, except chromosome 1A. The total number of markers was 9,267, of which 4,033 were on the A genome and 5,594 were on the B genome. The number of markers per chromosome varied from 162 (chromosome 4B) to 1,217 (chromosome 6B). The map length spanned 2,119.2 cM, as 965.5 cM for the A genome, and 1,153.7 cM for the B genome.

QTL analysis was performed using the composite interval mapping method (Zeng, 1994) with the Qgene software, version 4.3.10 (Joehanes and Nelson, 2008). A simplified, but representative, version of the linkage map with a total of 1,627 non-co-mapping markers was used to speed up the computation time. A scanning interval of 1 cM between markers and putative QTLs was used, with a window size of 10 cM, to detect the QTLs. Marker cofactors for the background control were set by single marker regression and simple interval analysis, with a maximum of five controlling markers. Putative QTLs were defined as two or more linked markers that were associated with a trait at a logarithm of the odds ratio (LOD) equal to or >3. Suggestive QTLs at the sub-thresholds as 2.0 < LOD < 3.0 values were also considered only where they co-localized with one or more statistically significant QTLs. For the main QTL effects, the positive sign of the estimates indicates that the “Ofanto” allele contributed toward higher values for the trait. The intervals of the QTLs and the flanking markers were determined following the method described by Darvasi and Soller (1997). The proportion of phenotypic variance explained (PVE) by a single QTL was determined by the square (*R*^2^) of the partial correlation coefficient. Graphical representations of the linkage groups were carried out using the MapChart 2.2 software (Voorrips, 2002). Candidate genes were searched in the chromosomal interval of the major QTLs identified in the present study. The sequences of the DArTseq markers (around 70 bp) within the estimated interval of each QTL were used as queries in a BLAST search against the bread wheat genome in the EnsemblPlants website with default parameters, except the “short sequences” option in the “search sensitivity” field, (https://plants.ensembl.org/Triticum_aestivum/Tools/Blast?db=core). The output of this search was the scaffold corresponding to each marker, and for each scaffold the physical and the genetic position were available (Clavijo et al., 2017). These positions were compared to those obtained in the same way for a number of candidate genes retrieved in the literature for the traits considered in the present study. In particular, the sequences of candidate genes were retrieved in the Gene Bank through the NCBI website (https://www.ncbi.nlm.nih.gov/) with the accession numbers and gene names reported in literature (see references for each gene in the Results section).

## Results

### Phenology

In the two experiments with May sowing, the daylength was the maximum natural daylength for the latitude of this Mediterranean environment (i.e., long day; LD; Table 1), whereas all the other experiments had December sowing where the plants were grown under short-day conditions (SD). The thermal environment was highly variable across the conditions, not only as a consequence of the various sowing/transplanting dates, but also because three of the experiments were performed in greenhouses (i.e., SD-V, SD-V-IN, SD-NV-IN) and the others outdoors (i.e., LD-V, LD-NV, SD-FI, SD-V-OUT, SD-NV-OUT). This also resulted in variations in the radiative environment between the greenhouse and outdoors, as the photosynthetically active radiation within the greenhouse was about half that recorded outdoors.

The eight conditions/ environments compared had large effects on the length of the transplanting–anthesis period (Table 2), which varied from 42 to 147 days. The shorter transplanting–anthesis periods were during the long-day studies and were mainly attributable to the short phyllochron (<80 °Cd). In response to the artificial vernalization, about two leaves less were produced (FLN, 11 vs. 13, *P* < 0.0001). A detailed discussion of the phenological data relative to these LD and SD-V studies was given in Sanna et al. (2014).

**Table 2:** Phenological traits recorded for the eight conditions (based on the eight lines in common).

**Treatment code^a^**	**Anthesis**			**Final leaf Number (*n*)**	**Phyllochron (°Cd)**
	**Date**	**Days after transplanting**	**(°Cd)**		
LD-V	5 July	42 ± 1	814	8.9 ± 0.2	74 ± 2
LD-NV	26 July	63 ± 4	1,291	13.6 ± 0.7	78 ± 3
SD-V	9 April	107 ± 1	1,626	12.2 ± 0.2	116 ± 1
SD-FI	7 May	147 ± 1	1,571	11.7 ± 0.2	98 ± 1
SD-V-IN	2 April	106 ± 2	1,560	12.9 ± 0.3	109 ± 2
SD-NV-IN	7 April	111 ± 2	1,638	11.9 ± 0.1	112 ± 2
SD-V-OUT	3 May	137 ± 1	1,637	12.4 ± 0.4	113 ± 2
SD-NV-OUT	3 May	137 ± 1	1,638	11.5 ± 0.4	129 ± 3

*Data are means ± standard error*.

a *See Table 1 for details*.

### Quantitative trait loci mapping and phenology

In general, the QTLs associated with the phenological traits were characterized by higher LOD scores, and they had higher PVE than the tillering and fertility traits (Table 3). The inclusion of the field study in addition to the three pot experiments of Sanna et al. (2014), and the updated genetic map used here with respect to that used by Sanna et al. (2014), resulted in the detection of more QTLs for developmental traits than in Sanna et al. (2014). The most important among these new QTLs (PVE > 20%) that were identified in these four environments according to the phenological traits were mainly located on chromosomes of groups 1, 2, 3, and 7 (Figure S1). Three relevant new QTLs were detected under the LD-V conditions: QTL 19 on linkage group 3B-1 associated to ANT (PVE = 20%), and two other QTLs (i.e., 4, 58) associated to FLN with PVE 30 and 20%, respectively. The lack of artificial vernalization under the long-day conditions resulted in the mapping of QTL 33, as QTL 10 previous described by Sanna et al. (2014), with the new map giving higher LOD scores and PVE (up to 59% for ANT and 47% for PHY). Three new QTLs for PHY were identified in SD-grown plants, as two on chromosome 2B (QTL 10, PVE = 21%; QTL 15, PVE = 20%) and one on chromosome 7A-1 (QTL 50, PVE = 28%). Three other QTLs were detected for the SD-V plants, associated to FLA and ANT (QTL 11) and FLN (QTLs 22, 50). The new SD-FI conditions resulted in detection of QTL 16 for FLA, QTL 58 for ANT, and QTL 10 associated to FLA and ANT as well as PHY. This last (i.e., QTL 10) corresponded to QTL 4 of Sanna et al. (2014), although with higher LODs and PVE (FLA, 40%; ANT, 69%). In general, under the SD conditions either in the greenhouse or in the field, the contributions of the chromosomal regions around the *Ppd-B1* locus (i.e., linkage group 2B-1) were demonstrated, even if the peak markers and the relative QTLs associated with ANT and FLA were different.

**Table 3:** Quantitative trait loci identified for each condition, their position relative to each linkage group, their confidence interval, LOD, PVE, and additive effect.

**QTL**	**Sanna et al., 2014**	**Candidate genes**	**Linkage group**	**Position (cM)**	**CI**	**Peak marker**	**LD-V**	**LD-NV**	**SD-V**	**SD-FI**	**LOD**	**PVE (%)**	**Add. Eff**.
1	*QTL1*		1B-1	36	9.5	wPt-2389				PHY	4.1	18.0	1.56
2			1B-2	12	13.3	4260609			K/SPIKEtill		2.9	12.9	1.49
2			1B-2	14	14.8	1229574				FLN	2.4	11.6	0.11
3			1B-2	23	9.2	1101782			K/SPIKEtill		4.2	18.6	−1.99
4			1B-2	63	5.8	3023789	FLN				6.8	29.6	−0.25
5			2A-2	16	12.8	4009855		ANT			2.8	13.4	57.83
5			2A-2	16	10.3	4009855		FLA			3.5	16.6	61.60
5			2A-2	16	9.7	4009855		FLN			3.8	17.7	0.64
5			2A-2	16	12.3	4009855		LN_TS_			3.1	13.9	0.31
6		*Vrs1, CEN, SUSIBA2*	2A-1	11	9.6	5567425			FERTILL		4.0	17.8	0.23
6			2A-1	16	12.7	5332093			CHAFFms		3.0	13.5	0.04
6	*QTL 15*		2A-1	23	15.1	3025308		FLN			2.3	11.4	0.52
6	*QTL 15*		2A-1	24	11.1	3945193		LN_TS_			3.5	15.5	0.60
6	*QTL 15*		2A-1	31	13.9	3959527		FERTILL			2.7	12.3	0.15
7			2A-1	86	10.2	3941252	K/CHAFFtill				3.8	16.9	−1.53
7			2A-1	89	14.4	980420			ANT		2.4	11.9	−22.57
8			2A-1	114	12.5	3532866	K/SPIKEms				3.0	13.7	−1.93
8			2A-1	116	16.7	1042688				ANT	2.1	10.3	6.81
9			2B-2	28	10.2	1213316				K/CHAFFms	3.8	16.8	1.57
9			2B-2	30	15.1	3937704				ANT	2.3	11.4	−13.71
9			2B-2	30	9.6	3937704			ANT		3.8	17.9	−28.02
9			2B-2	30	12.9	3937704			FLA		2.8	13.3	−22.67
9			2B-2	50	9.3	4406008		CHAFFtill			4.2	18.5	0.01
10	*QTL 4*	*Ppd-B1*	2B-1	0	13.4	wPt-5788	LN_TS_				2.8	12.8	−0.09
10	*QTL 4*		2B-1	2	4.2	3934592				FLA	10.0	40.4	−29.80
10	*QTL 4*		2B-1	2	14.5	3934592		ANT			2.4	11.8	−33.70
10			2B-1	4	12.4	4410465			K/CHAFFtill		3.1	13.8	2.84
10			2B-1	8	2.5	5577186				ANT	22.4	68.7	−31.54
10			2B-1	8	8.2	5577186				PHY	4.8	20.9	−1.72
10			2B-1	8	14.3	5577186			FLN		2.5	12.0	−0.13
11			2B-1	18	13.7	1020393		SPKLTms			2.8	12.5	0.16
11			2B-1	20	3.8	3028596			ANT		11.5	44.7	−38.54
11			2B-1	20	7.1	3028596			FLA		5.4	24.3	−30.19
11			2B-1	20	13.6	3028596		K/SPKLTms			2.8	12.6	−0.08
11			2B-1	22	9.7	wPt-5556			CHAFFtill		4.0	17.6	−0.03
12		*Constans4, Vers1, CEN, SUSIBA2*	2B-1	60	10	3533677		K/CHAFFtill			4.1	18.0	−4.89
13			2B-3	0	12.2	5325236	K/SPIKEtill				3.1	14.1	0.11
13			2B-3	2	14.9	2249524			ANT		2.4	11.5	24.67
13			2B-3	2	33.6	2249524			FLA		1.0	5.1	15.43
14			2B-3	32	9.3	4410489	K/SPIKEms				4.2	18.5	0.92
14			2B-3	42	6.7	wPt-7004		K/SPIKEtill			6.2	25.8	0.92
14			2B-3	44	11.9	1240435			HS_MAXTILL_		3.2	14.4	−0.37
15			2B-3	62	12.3	1258509	PHY				3.1	14.0	0.49
15			2B-3	68	8.5	1109533			PHY		4.5	20.3	2.46
16		*HKX9, TaGw2*	3A-1	3	11	5371026				K/SPKLTms	3.4	15.0	−0.03
16			3A-1	15	6.9	1088186				FLA	5.6	25.0	−20.46
16			3A-1	20	10.4	1166451			K/SPIKEtill		3.7	16.5	−1.37
16			3A-1	21	17.2	4009170	FLN				2.0	10.0	0.20
16			3A-1	28	10.2	1089657			CHAFFtill		3.8	16.9	−0.03
16			3A-1	28	13.3	1089657		ANT			2.7	12.9	58.14
16			3A-1	28	10.9	1089657		FLA			3.3	15.8	40.78
17			3A-1	83	12.2	4992623	FERTILL%				3.1	14.1	−2.01
18			3A-2	0	11.2	5345498			K/CHAFFtill		3.4	15.3	−2.39
18			3A-2	4	7.8	1222082		K/CHAFFtill			5.1	22.0	−3.94
19			3B-1	1	8.7	1018357	ANT				4.2	19.7	−84.95
20		*FT2, BRI1, ERA1*	3B-1	30	14	1669818		RATE			2.6	12.0	−0.04
20			3B-1	34	9.9	4540162		FERTILL			4.0	17.4	−0.19
20			3B-1	34	8.8	4540162		MAXTILL			4.5	19.5	−0.64
21			3B-1	83	9.7	1735063		SPKLTms			4.0	17.7	0.20
21			3B-1	90	11.9	3533183				K/SPKLTms	3.2	14.4	−0.03
21			3B-1	91	16.3	4005415	ANT				2.1	10.5	13.98
21			3B-1	97	14.3	3222436			FLN		2.5	12.0	0.13
21			3B-1	99	9.2	4411657		ANT			4.0	18.6	−43.00
21			3B-1	107	13.7	2259639		FLN			2.6	12.5	−0.55
21			3B-1	111	12.2	1093348		CHAFFms			3.1	14.1	0.02
21	*QTL 5*		3B-1	113	12.5	5567618	CHAFFms				3.0	13.7	0.02
21	*QTL 5*		3B-1	122	12.5	1006229		FLA			2.8	13.7	−56.23
22	*QTL 6*		3B-1	147	6.2	4004851			FLN		6.2	27.6	0.22
23			3B-2	0	16.7	2267290				ANT	2.1	10.3	12.93
23			3B-2	0	12.7	2267290				FLA	2.8	13.5	20.09
23			3B-2	0	11.7	2267290			LN_TS_		3.3	14.7	−0.10
23			3B-2	0	12.5	2267290	K/SPIKEms				3.0	13.7	0.73
23			3B-2	8	11.1	1060402	CHAFFms				3.4	15.4	0.02
24			4A-3	23	10.5	4008720				FLN	3.4	16.3	−0.14
24			4A-3	23	11.7	4008720	LN_TS_	ANT			3.1	14.7	−64.63
24			4A-3	23	9.6	4008720	LN_TS_				4.0	17.8	−0.11
24			4A-3	23	15.9	4008720		FLA			2.2	10.8	−53.14
24			4A-3	23	13.8	4008720		FLN			2.6	12.4	−0.57
25			4A-1	13	11.3	1115744			CHAFFtill		3.4	15.2	0.03
26			4A-4	56	10.1	3024608		K/SPIKEms			3.8	17.0	−3.17
26			4A-4	56	12.5	3024608		LN_TS_			3.0	13.7	−0.31
27			4A-4	90	13.7	5010723	FERTILL				2.8	12.5	−0.06
27			4A-4	90	12.3	5010723	K/CHAFFtill				3.1	14.0	−1.40
28			4B	0	10.7	1153087			FLN		3.4	16.0	−0.18
28			4B	2	14.3	1161417	FERTILL				2.6	12.0	0.06
28			4B	4	11.8	3952452			CHAFFms		3.3	14.6	−0.06
28			4B	14	12.3	1153087	RATE				3.1	14.0	0.03
28			4B	18	13.8	Rht-B1		K/SPKLTms			2.7	12.4	−0.16
28			4B	26	15.3	Rht-B1			LN_TS_		2.5	11.2	0.12
29	*QTL7*		4B	44	8.1	Xgwm710			FERTILL		4.9	21.2	0.33
29	*QTL7*		4B	44	8.1	Xgwm710			MAXTILL		4.9	21.2	0.34
29	*QTL7*		4B	46	15.1	wPt-4931				FLN	2.3	11.4	0.14
30			4B	160	13.3	4537971	K/SPIKEms				2.9	12.9	−0.69
30			4B	172	9.6	5567462	SPKLTms				4.1	17.9	−0.26
31			5A-1	12	15.6	1007490			RATE		2.4	11.0	0.01
31			5A-1	12	12.6	1007490		K/SPKLTms			3.0	13.6	−0.09
31			5A-1	16	9.2	1003869		K/SPIKEms			4.3	18.6	−2.32
31	*QTL8*		5A-1	27	13.6	4005607				CHAFFms	2.8	12.6	−0.04
32	*QTL9*		5A-1	62	11.8	4412180	CHAFFms				3.2	14.5	−0.02
32	*QTL9*		5A-1	62	7.9	4412180	CHAFFtill				5.0	21.6	−0.04
33			5A-2	20	6.0	1072802		HS_MAXTILL_			6.9	28.6	−0.15
33			5A-2	22	3.7	1241769		PHY			12.6	46.5	−2.34
33	*QTL 10*		5A-2	24	2.9	5567501		ANT			17.1	58.7	−112
33	*QTL 10*		5A-2	24	7.2	5567501		CHAFFms			5.6	23.7	0.03
33	*QTL 10*		5A-2	24	12.1	5567501		CHAFFtill			3.1	14.2	0.01
33	*QTL 10*		5A-2	24	3.3	5567501		FLA			14.1	51.7	−95.56
33	*QTL 10*		5A-2	24	3.7	5567501		FLN			12.3	47.0	−1.00
33	*QTL 10*		5A-2	24	4.1	5567501		LN_TS_			11.1	41.5	−0.48
33	*QTL 10*		5A-2	24	5.6	5567501		MAXTILL			7.6	30.8	−1.44
33	*QTL 10*		5A-2	32	8.8	4910545			K/CHAFFms		4.4	19.4	2.76
33	*QTL 10*		5A-2	36	11.9	3028086	PHY				3.2	14.4	0.50
33	*QTL 10*		5A-2	39	5.3	1200696		FERTILL			8.1	32.5	−0.30
33	*QTL 10*		5A-2	39	8.3	1200696		FERTILL%			4.8	20.6	−1.37
33	*QTL 10*		5A-2	39	7.8	1200696		K/CHAFFms			5.1	22.1	−4.28
33	*QTL 10*		5A-2	40	11.5	1229087		K/CHAFFtill			3.3	14.9	−2.84
34			5A-2	82	12.1	1200768		CHAFFtill			3.2	14.2	0.01
35			5A-2	124	10.8	2261896	FLN				3.3	15.9	−0.25
36			5A-3	10	10.5	2280261			ANT		3.4	16.3	20.91
37			5B-1	20	14.7	3959042			K/SPIKEtill		2.6	11.7	−1.05
37			5B-1	22	12.8	1072798				K/SPKLTms	3.0	13.4	−0.03
37			5B-1	22	16.8	1072798	FLA				2.1	10.2	13.50
37			5B-1	22	9.1	1072798	K/SPKLTms				4.3	18.8	−0.05
37			5B-1	30	17.2	1267868				ANT	2.0	10.0	6.93
38			5B-1	54	10.0	4407395			PHY		3.8	17.2	3.96
39			5B-3	2	9.1	2276466			K/CHAFFtill		4.3	18.9	−4.95
39			5B-3	2	9.5	2276466	K/CHAFFtill				4.1	18.0	−1.59
40			5B-2	2	13.7	1130110			FERTILL		2.8	12.5	0.19
40			5B-2	4	10.0	wPt-0054			MAXTILL		3.9	17.1	0.26
40			5B-2	6	14.5	wPt-7665			RATE		2.6	11.8	0.01
41		*TaGw2*	6A	27	10.1	1105205				CHAFFms	3.8	17.0	−0.16
41			6A	31	5.7	1025005				K/CHAFFms	7.4	30.2	2.36
42			6A	64	12.5	3934210			K/SPKLTms		3.1	13.7	0.07
42			6A	75	10.5	4394087			ANT		3.4	16.3	−21.17
43			6A	87	12.9	3953338		K/CHAFFms			2.9	13.3	3.25
43			6A	87	12.6	1070905		PHY			2.9	13.6	1.02
44			6A	105	10.3	1408556			K/SPIKEms		3.8	16.6	2.07
45			6B-1	17	7.5	1104197			HS_MAXTILL_		5.4	22.9	−0.51
46			6B-2	31	6.6	Xgwm963		FERTILL			6.2	26.0	0.24
46			6B-2	34	13.9	4993794		FERTILL%			2.7	12.3	0.92
47			6B-2	59	10.6	1123289				K/SPIKEms	3.6	16.2	−2.28
47			6B-2	61	11.2	3942467				FLA	3.2	15.3	22.32
47			6B-2	69	11.0	5325371				SPKLTms	3.5	15.6	−0.24
48			6B-2	84	15.5	3532795		ANT			2.3	11.1	54.01
48			6B-2	87	11.6	1699040			FERTILL		3.3	14.8	−0.22
48			6B-2	87	11.2	1699040			MAXTILL		3.4	15.3	−0.23
48			6B-2	87	9.7	1699040	SPKLTms				4.0	17.6	−0.23
48			6B-2	90	9.1	1769376			HS_MAXTILL_		4.3	18.9	−1.05
49			6B-2	125	9.7	1275751			FERTILL		4.0	17.6	0.24
49			6B-2	125	15.5	1275751			MAXTILL		2.4	11.1	0.19
49			6B-2	134	13.2	5580299		PHY			2.8	13.0	0.96
50			7A-1	0	9.3	1128723				K/CHAFFms	4.2	18.4	1.67
50			7A-1	4	8.8	1019140			FLN		4.2	19.5	0.19
50			7A-1	10	7.3	5353667				ANT	5.2	23.6	−11.40
50			7A-1	12	8.9	4410356				FLA	4.1	19.2	−17.05
50			7A-1	12	6.1	4410356				PHY	6.8	28.2	−2.03
50			7A-1	12	11.6	4410356		MAXTILL			3.3	14.8	0.54
51			7A-1	46	10.7	3955101				CHAFFms	3.6	16.0	0.04
51			7A-1	51	14.2	Xgwm276			FLN		2.5	12.1	−0.13
51			7A-1	51	10.3	Xgwm276	FLN				3.5	16.7	0.18
51			7A-1	54	9.5	4992699		FERTILL			4.1	18.0	0.20
51			7A-1	61	9.4	1236907		FERTILL%			4.2	18.2	1.51
52			7A-1	78	6.7	1212458	SPKLTms				6.1	25.5	−0.30
52			7A-1	82	13.7	1215067				K/SPKLTms	2.8	12.5	0.03
53		*DWARF3*	7A-4	18	8.1	5368531			CHAFFms		4.9	21.3	−0.05
54			7B-1	18	13.6	1018944	FLA				2.6	12.6	−15.66
54			7B-1	20	14.4	1249253	ANT				2.4	11.9	−18.95
54			7B-1	30	9.5	4003174	CHAFFms				4.1	18.1	−0.02
54			7B-1	32	13.5	1195791			CHAFFms		2.8	12.7	−0.04
54			7B-1	32	9.3	1195791	FERTILL				4.2	18.4	0.07
54			7B-1	34	11.6	3949256	SPKLTms				3.3	14.8	−0.29
55	*QTL14*	*Constans6*	7B-1	74	13.2	1097741		CHAFFtill			2.9	13.0	−0.01
55	*QTL14*		7B-1	80	11.1	4993641	FLA				3.2	15.5	−17.75
55			7B-1	82	8.4	1772284			SPKLTms		4.7	20.4	−0.74
55			7B-1	84	11.1	5011372		SPKLTms			3.5	15.5	−0.22
55			7B-1	90	4.6	1113703	FLN				9.0	37.3	−0.34
55			7B-1	90	13.0	1121517	HS_MAXTILL_				2.9	13.2	−0.07
55			7B-1	90	13.7	1121517	MAXTILL				2.8	12.5	−0.19
55			7B-1	94	9.2	1113456		K/SPIKEtill			4.3	18.6	−0.78
55			7B-1	100	9.7	Xgwm783			HS_MAXTILL_		4.0	17.6	0.43
56			7B-2	14	11.4	5411613			SPKLTms		3.4	15.1	0.55
57			7B-2	32	13.6	1264692	FLN				2.6	12.6	−0.23
57			7B-2	40	16.8	5347028			ANT		2.1	10.2	−22.09
57			7B-2	40	8.9	5347028			FLA		4.1	19.3	−23.15
58			7B-3	0	6.4	1065475				ANT	6.1	27.0	−43.06
58			7B-3	0	16.0	1065475	ANT				2.2	10.7	−17.42
58			7B-3	0	6.3	1065475	FLA				6.1	27.1	−18.96
58			7B-3	0	8.4	1065475	FLN				4.4	20.4	−0.27
58			7B-3	0	5.3	1065475	SPKLTms				8.1	32.4	−0.35
58			7B-3	4	16.5	5374057		ANT			2.1	10.4	−31.53
59			7B-3	26	11.8	3958430			FERTILL%		3.2	14.6	0.88
59			7B-3	34	11.2	4406269			FERTILL		3.4	15.3	0.06

### Tillering phenotyping

**Figure 1:**
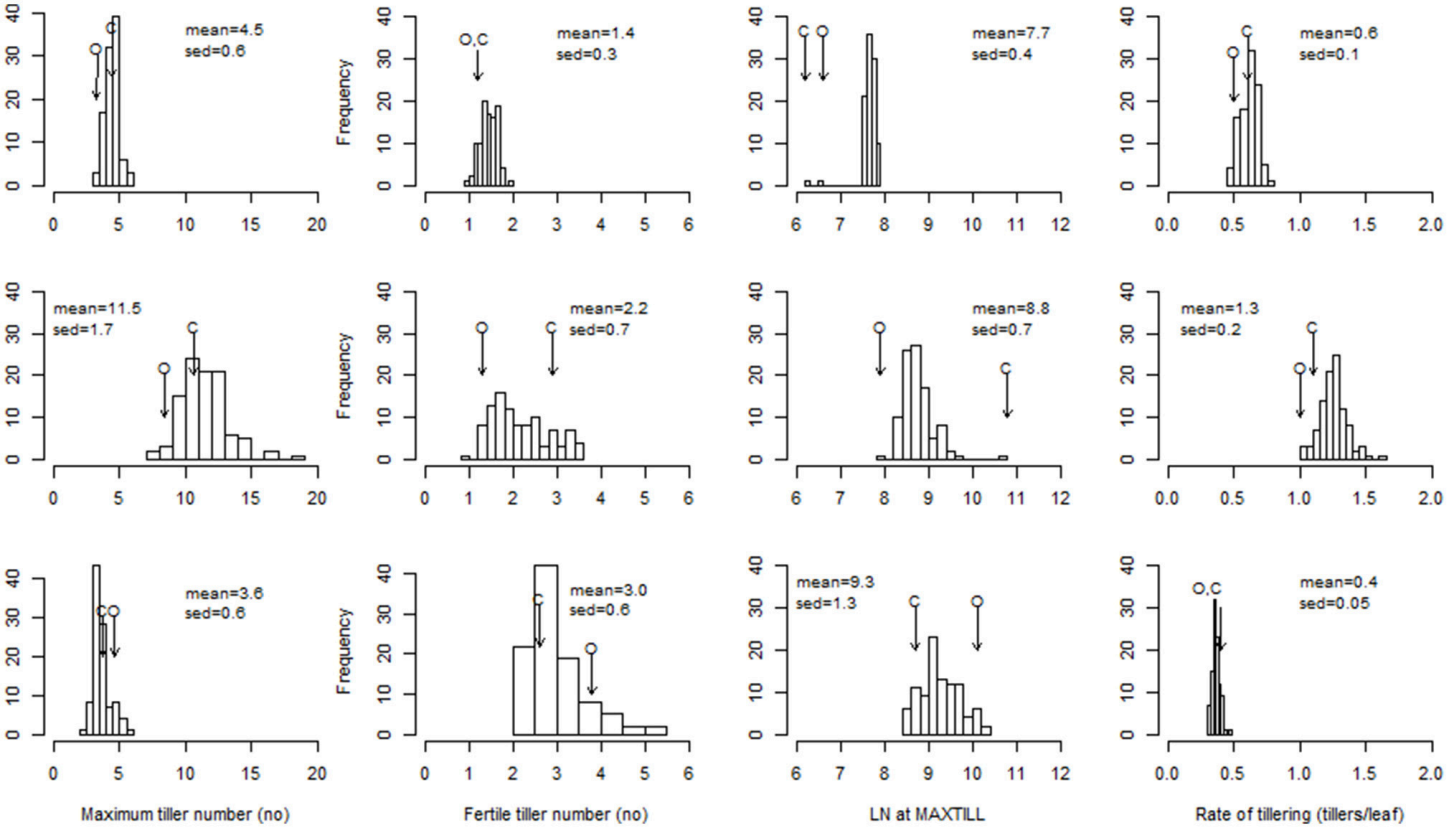
Distribution of the ril best linear unbiased predictors (BLUPs) for maximum tiller number, fertile tiller number, leaf number at MAXTILL, and rate of tillering. Top row to bottom row: LD-V plants, LD-NV plants, SD-V plants. Arrows indicate the performance of the parents (O: cv. “Ofanto”; C: cv. “Senatore Cappelli”). sed: standard error of the difference between BLUPs.

The ANOVA derived from the full dataset indicated the absence of any main genetic effects for most of the tillering traits, and the presence of a major genotype × treatment interaction (Table S1). The range of variability between the RILs for MAXTILL and RATE was highest under the LD-NV condition (11 tillers; 0.6 tillers per leaf, respectively; Figure 1). This large genetic variability reflected transgressive segregation, as the differences between the parents were much smaller than the range of RILs. The distributions of the RILs for the tillering traits were normal or skewed toward smaller values.

The comparison between the LD-V and LD-NV environments allowed estimation of the effects of vernalization on the tillering traits. Absence of vernalization defined higher MAXTILL (11.5 vs. 4.5), which was derived from both higher LN_MAXTILL_ (8.8 vs. 7.7 leaves) and higher RATE (1.3 vs. 0.6 tillers per leaf; Figure 1). A rate of tillering >1.0 means that tillers of order >1.0 were produced. This can explain why this effect of vernalization on the MAXTILL was reduced when the FERTILL numbers (i.e., tillers bearing a spike at maturity) were compared (1.96 vs. 1.42). Most of the secondary and tertiary tillers did not survive to produce a spike, and the proportion of sterile tillers increased at the higher MAXTILL numbers of the NV environment (from 73 to 83%). On the other hand, these high percentages of sterile tillers cannot be attributed to inter-plant competition for resources, as these plants were grown in pots with optimal nutrient and water availability and without any competition for radiation. In any case, a genetic correlation was observed within each environment between MAXTILL and FERTILL (*r* = 0.31, *P* ≤ 0.01 in LDV; *r* = 0.58, *P* ≤ 0.001 in LDNV; *r* = 0.89, *P* ≤ 0.001 in SDV).

These effects of vernalization were also analyzed in the “subsets” shown in Table 4, with the aim being to determine whether the vernalization effects on tillering observed in the population under the long-day conditions and the relatively high temperatures of May and June were also evident under the short-days and the different thermal conditions. This subset confirmed the positive effects of the lack of vernalization on tillering. The non-vernalized RILs produced a mean of 9.6 ± 0.3 tillers, vs. 7.4 ± 0.2 produced by the vernalized RILs. However, this difference was derived solely from the higher number of tillers per leaf produced without vernalization, as LN_MAXTILL_ was the same. The smaller, although still significant, difference in MAXTILL observed in this case between vernalization and non-vernalization can be attributed to some natural vernalization of the NV plants, as the temperatures were lower than those of the LD conditions (Table 1) due to the different thermal environments of the different sowing dates. The tiller mortality or sterility was of the same order of magnitude to that observed for the long days, which lead to a lack of any effects of vernalization on the number of fertile tillers.

**Table 4:** Tillering traits recorded for the subsets, and results of ANOVA.

**Treatment code^a^**	**MAXTILL (*n*)**	**FERTTILL (*n*)**	**LN_MAXTILL_ (*n*)**	**RATE (tillers/leaf)**
SD-NV-IN	9.7 ± 0.5	1.8 ± 0.14	9.6 ± 0.03	1.01 ± 0.05
SD-V-IN	7.5 ± 0.3	1.6 ± 0.11	9.7 ± 0.03	0.77 ± 0.03
SD-NV-OUT	9.4 ± 0.4	1.7 ± 0.10	8.9 ± 0.06	1.06 ± 0.05
SD-V-OUT	7.4 ± 0.2	1.7 ± 0.12	8.7 ± 0.06	0.84 ± 0.03
Vernalization	<0.0001	ns	ns	<0.001
IN vs. OUT	ns	ns	<0.001	ns

a *See Table 1 for details*.

Cultivar “Senatore Cappelli” was the parent with the highest MAXTILL and FERTILL under all of the non-vernalized conditions (including the subsets). This was not a consequence of the *Rht* genes, because the comparison of the two groups of 100 RILs of different genotypes at the *Rht-B1* locus showed that their differences in MAXTILL and FERTILL were not significant.

The effects of temperature on tillering were evaluated in the subsets by comparing plants grown in the greenhouse (Table 4, IN) with those grown in the same period outdoors (Table 4, OUT). These data show that the higher temperatures inside the greenhouse only increased LN_MAXTILL_ (from 8.8 ± 0.04 to 9.6 ± 0.02), without any effects on MAXTILL.

The genetic correlations between the tillering and phenological traits reported in Table 5 clearly show that the lack of vernalization resulted in the strongest relationships between phenology and tillering traits. In contrast, the limiting photoperiod conditions of SD-V had almost no effects on the establishing of any relationships between phenology and tillering. A role of the *Eps* genes on tillering was also apparent, as most of the phenological traits were correlated with most of the tillering traits in the LD-V plants.

**Table 5:** Genetic correlations among the measured tillering and phenological traits for the long day vernalized and non-vernalized plants, and the short day vernalized plants.

**Treatment code^a^**	**Trait**	**MAXTILL**	**FERTILL**	**LN_MAXTILL_**	**RATE**
LD-V	FLN	**0.49**	ns	**0.44**	**0.37**
	LN_TS_	**0.44**	ns	0.24	**0.38**
	PHY	ns	ns	−**0.43**	ns
	ANT	**0.33**	−0.25	ns	**0.35**
LD-NV	FLN	**0.53**	**0.60**	**0.58**	0.25
	LN_TS_	**0.49**	**0.50**	**0.54**	0.29
	PHY	0.26	0.31	0.24	0.26
	ANT	**0.40**	**0.53**	**0.48**	0.22
SD-V	FLN	ns	ns	0.21	ns
	LN_TS_	0.33	0.28	0.26	0.20
	PHY	ns	ns	−0.22	ns
	ANT	ns	ns	ns	ns

a *See Table 1 for details. ns, not significant, normal text, significant at P ≤ 0.05, bold text, significant at P ≤ 0.01*.

The only phenological trait that was always associated with tillering was LN_TS_, which affected MAXTILL, RATE, and LN_MAXTILL_ in all of the environments. This indicates that the higher the LN_TS_, the longer the period available for tillering (i.e., expressed as number of leaves produced during tillering), the higher the number of tillers per leaf, and ultimately, the higher the MAXTILL. A high LN_TS_ can derive from the long duration of the sowing–TS period and/or from a high rate of leaf primordia production due to high temperatures. In both cases, the increased number of leaves at TS represented more potential tillering sites. Time to anthesis and FLN affected tillering in the two long-day environments, but not under the SD-V conditions, when the photoperiod alone was controlling development, because in this case genetic variability in LN_TS_ was not related to anthesis. PHY had only a marginal impact on tillering.

### Tillering QTLs

For the tillering traits, this study identified a total of 33 QTLs, where nine of these explained phenotypic variance >20% (Table 3). Under the SD-V conditions, where the *Ppd* genes were active, the QTLs associated with the LN_MAXTILL_, FERTILL and MAXTILL traits were located on chromosomes 6B (QTLs 45, 48) and 4B (QTL 29), respectively. Under these conditions, although the region on chromosome 2B that was close to the *Ppd-B1* locus was very active in its effects on the duration of the main phenological stages of the crop, it did not affect tillering.

In contrast, when the *Vrn* genes were active (i.e., under the SD-NV conditions), the association with the major QTL 33 located on the linkage group 5A-2 in the same region as the *Vrn-A1* locus was highly significant for all tillering traits (from 21 to 33%), except for RATE. QTL 33 was also associated with all of the phenological traits examined, and in all cases it explained phenotypic variation >40%. Under these conditions (i.e., SD-NV), also the effects of QTL 46 (chromosome 6B) on FERTILL (Xgwm963) and QTL 20 (chromosome 3B) on MAXTILL were significant.

On the contrary, under the conditions in which the *Eps* genes were active (i.e., LD-V), the only significant associations identified were those for RATE on chromosome 4B (QTL 28) and for FERTILL on chromosome 7B (QTL 54). RATE was the main determinant of MAXTILL under the LD-V conditions, and the chromosomal regions identified for this trait flanked the *Rth-B1* locus on chromosome 4B (QTLs 28, 29). Accordingly, when the RILs were grouped based on their genotypes at the *Rht-B1* locus, significant difference was detected for MAXTILL under the LD-V conditions (4.08 vs. 4.55, *P* < 0.05). The apparent effect of the *Eps* genes on tillering traits that was highlighted by the genetic correlations was not confirmed by the molecular analysis.

### Fertility phenotyping

**Figure 2:**
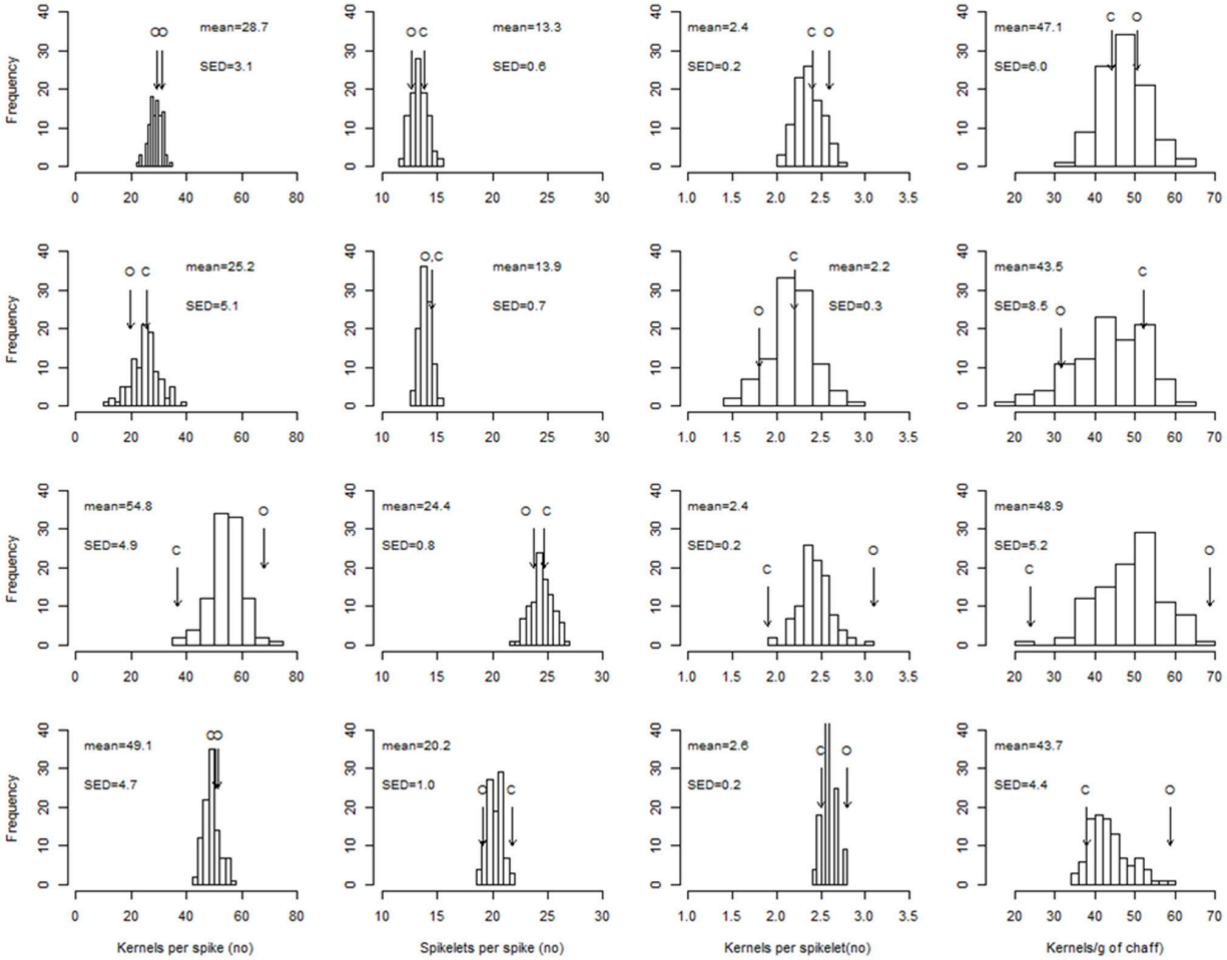
Distribution of the RIL best linear unbiased predictors (BLUPs) for the number of kernels per spike, spikelets per spike, kernels per spikelet, and kernels per g chaff. Top row to bottom row: LD-V plants, LD-NV plants, SD-V plants, SD-FI plants. Arrows indicate the performance of the parents (O: cv. “Ofanto”; C: cv. “Senatore Cappelli”). SED: standard error of the difference between BLUPs.

The SD-V conditions provided the environment where there was the largest range of variability between the RILs (Figure 2) in fertility traits, and where the highest heritabilities within the environment (Table S2) were observed. For most of the traits, the large genetic variability of the SD-V–grown RILs was not reflected in transgressive segregation, as the differences between the parents were almost as large as the population range (Figure 2). In general, environmental conditions induced a higher variation in SPKLT (i.e., from means of 13 for LD-V to 24 for SD-V) compared to genotype (i.e., from 2.5 to 5.3 under the SD-V conditions).

According to the mean population values, the short-day conditions had positive effects on both SPKLT (i.e., from 13.3 to 24.4 with the vernalized plants) and K/SPIKE (i.e., from 28.7 to 54.8 with the vernalized plants), although the short days of the December sowing and the long days of the May sowing also differed greatly for the thermal conditions (16.6 vs. 20.4°C, respectively, in the sowing–anthesis period). To reduce this difference in temperature, the plants grown in the greenhouse in the SD-V-IN subset (i.e., 18.4°C in the sowing–anthesis period; Table 6) were compared with the same subset of lines extracted from the LD-V (data not shown). SPKLT was still significantly higher under the short days (23.7 vs. 13.4, *P* < 0.001), as was K/SPIKE (49.0 vs. 32.4, *P* < 0.001) and CHAFF (1.57 vs. 0.64 g spike^−1^, *P* < 0.001), whereas K/CHAFF was lower (32 vs. 51 grains g^−1^, *P* < 0.001). The subsets supported the positive effect of the higher temperatures recorded in the greenhouse compared to outdoors seen for SPKLT (23.8 ± 0.2 vs. 20.4 ± 0.4) and CHAFF (1.44 ± 0.06 vs. 1.04 ± 0.04). In contrast to the daylength effect, higher SPKLT was not accompanied by higher K/SPIKE, because of the negative effect of the greenhouse conditions on K/SPKLT (2.2 ± 0.1 vs. 2.5 ± 0.1). A higher K/CHAFF was observed outdoors (44.8 ± 2.0) compared to in the greenhouse (33.1 ± 1.7), which was probably derived from the lower radiation in the greenhouse during spike growth, compared to outdoors.

**Table 6:** Fertility traits recorded for the subset for main stems and tillers, and results of ANOVA.

	**Treatment code^a^**	**SPKLT (*n*)**	**K/SPIKE (*n*)**	**K/SPKLT (*n*)**	**K/CHAFF (n g^−1^)**	**CHAFF (g spike^−1^)**
Main stem	SD-V-IN	23.7 ± 0.4	49.0 ± 3.6	2.3 ± 0.1	32.0 ± 2.6	1.57 ± 0.1
	SD-NV-IN	24.0 ± 0.3	43.9 ± 3.2	2.1 ± 0.1	34.1 ± 2.4	1.31 ± 0.0
	SD-V-OUT	20.2 ± 0.6	44.5 ± 1.8	2.4 ± 0.1	44.5 ± 2.5	1.04 ± 0.1
	SD-NV-OUT	20.6 ± 0.5	45.7 ± 1.7	2.5 ± 0.1	45.2 ± 3.2	1.05 ± 0.1
	Vernalization	ns	0.004	0.001	ns	ns
	IN vs. OUT	<0.001	ns	<0.001	<0.001	<0.001
Tillers	SD-V-IN		28.0 ± 2.2		51.4 ± 4.3	0.48 ± 0.0
	SD-NV-IN		32.4 ± 2.2		55.6 ± 4.1	0.54 ± 0.0
	SD-V-OUT		28.1 ± 1.7		47.7 ± 2.6	0.50 ± 0.0
	SD-NV-OUT		29.8 ± 1.2		52.5 ± 2.6	0.51 ± 0.0
	Vernalization		ns		ns	ns
	IN vs. OUT		ns		ns	ns

a *See Table 1 for details*.

The environmental association between SPKLT and K/SPIKE was also seen at the genetic level (Table 7), although in this case, K/SPKLT and K/CHAFF explained a greater proportion of K/SPIKE than SPKLT for all of the environments. The impact of phenology on fertility traits, and on SPKLT in particular, was maximum under the LD-V conditions, when the genetic variation in SPKLT was associated with the concomitant variation in FLN, ANT, and LN_TS_.

**Table 7:** Genetic correlations among the measured fertility and phenological traits for the long day vernalized and non-vernalized plants, and the short day vernalized and naturally vernalized plants.

**Treatment code^a^**	**Trait**	**SPKLT**	**K/SPIKE**	**K/SPKLT**	**K/CHAFF**	**CHAFF**
LD-V	K/SPIKE	0.31				
	K/SPKLT	ns	**0.83**			
	K/CHAFF	ns	**0.59**	**0.70**		
	CHAFF	**0.54**	0.29	ns	−**0.54**	
	FLN	**0.50**	ns	−0.21	−0.22	0.35
	LN_TS_	0.41	ns	ns	ns	ns
	PHY	ns	ns	ns	ns	ns
	ANT	**0.51**	ns	−0.23	−0.31	0.43
LD-NV	K/SPIKE	0.25				
	K/SPKLT	ns	**0.88**			
	K/CHAFF	ns	**0.71**	**0.74**		
	CHAFF	0.35	0.39	0.20	−0.30	
	FLN	ns	ns	0.27	0.44	−0.47
	LN_TS_	ns	ns	0.20	0.36	−0.40
	PHY	−0.22	ns	ns	0.35	−0.47
	ANT	ns	ns	ns	0.33	−**0.51**
SD-V	K/SPIKE	0.30				
	K/SPIKEKL	ns	**0.87**			
	K/CHAFF	ns	**0.59**	**0.66**		
	CHAFF	0.25	0.24	ns	−**0.57**	
	FLN	ns	ns	−0.28	−0.28	ns
	LN_TS_	ns	ns	ns	ns	ns
	PHY	ns	ns	ns	0.28	−0.30
	ANT	ns	ns	ns	ns	ns
SD-FI	K/SPIKE	**0.50**				
	K/SPIKEKL	ns	**0.76**			
	K/CHAFF	ns	ns	0.22		
	CHAFF	0.49	**0.59**	0.36	−**0.68**	
	FLN	0.31	ns	−0.38	−0.40	0.23
	PHY	0.30	ns	ns	−0.20	0.27
	ANT	ns	−0.27	−0.22	−0.20	ns

a *See Table 1 for details. ns, not significant, normal text, significant at P ≤ 0.05, bold text, significant at P ≤ 0.01*.

The same genes controlled the main stem and tiller fertility traits, as was seen by the close association between these (data not shown).

### Fertility QTLs

The number of significant QTLs associated with spike fertility and detected for the main stem (ms) and tillers (till) were 39 and 21, respectively, and were almost equally distributed among the environments. For SPKLTms, 11 QTLs were identified in the present study, three of which explained more than 20% of phenotypic variance and were located on chromosomes 7A and 7B (QTLs 58, 52, 55). All of the associations recorded for group 7 chromosomes for the SPKLTms trait overlapped with the FLN trait. In particular, SPKLTms and FLN shared the same peak marker 1065475 that was identified in LD-V plants for QTL 58 on chromosome 7B, and shared with QTL 55 on chromosome 7B, although with different peak markers. Then, on linkage group 7A-1, both of these traits reported a QTL closely associated in the same region. In addition, under the LD-V conditions, QTL 30 on chromosome 4B and QTL 48 on chromosome 6B were also identified.

For K/SPKLTms, eight QTLs were identified. One of these (i.e., QTL 37) showed a good PVE (19%), was localized on chromosome 5B with peak marker 1072798, and was co-located with the phenological trait FLA in the same environment (LD-V). Another QTL for K/SPKLTms (i.e., QTL 28 on chromosome 4B) had the *Rht-B1* gene as a peak marker, and explained 12% of the phenotypic variation in the fertility trait.

For K/SPIKEms, eight QTLs were identified located on eight different chromosomes, which explained up to 19% of its phenotypic variation. Of these, QTL 26 (PVE = 17%) shared the same peak marker with the phenological trait LN_TS_ in the LD-NV plants. K/SPIKEms also shared the same QTL 47 (PVE = 16%) with the phenological trait FLA for the SD-FI conditions. Of all of the QTLs for K/SPIKEms, only QTL 14 on linkage group 2B-3 showed a close association with the same trait detected for tillers (K/SPIKEtill). Apart from QTL 14, the chromosomal regions significantly involved in the expression of K/SPIKEtill were localized on linkage group 7B-1 (QTL 55) and 1B-2 (QTL 3).

For CHAFFms, 11 QTL were identified (nine with LOD >3), and those with a more marked effect were located on chromosomes 5A (QTL 33), 7A (QTL 53), 7B (QTL54), and 6A (QTL 41). Of these, QTL 54 overlapped with the QTL identified for SPKLT, FLN, and ANT, whereas QTL 33 (under the LD-NV conditions) and QTL 41 (under the SD-FI conditions) overlapped with two of the most important QTLs identified for K/CHAFFms. The association between CHAFFms and K/CHAFFms on QTL 41 and the opposite sign of their additive affects strongly support the negative genetic correlation (*r* = −0.68, *P* < 0.01) calculated between CHAFF and K/CHAFF under the SD-FI conditions. The analysis of tillers revealed seven QTLs associated with CHAFFtill, only two of which (i.e., QTLs 32, 33, on chromosome 5A) overlapped with those recorded for the main stem.

K/CHAFFms was associated with five QTLs, two of which (i.e., QTL 41 on chromosome 6A, QTL 33 on chromosome 5A) explained 30 and 22%, respectively, of its variation. QTL 41 was identified under the SD-V conditions, but QTL 33 was found under the NV conditions. This established a link between K/CHAFF and most of the phenological traits governed by the *Vrn* genes. The genetic correlation between K/CHAFF and K/SPIKLT seen for all of the environments was not confirmed at the molecular level, although QTL 41 co-located on the same chromosome (6A) at a relatively short distance, with QTL 42 associated with both K/SPIKLT and ANT under the SD-V conditions. Of the eight QTLs associated with K/CHAFFtill, only two QTLs were common to K/CHAFFms, while the rest (with greater PVE) were located on other chromosomal regions (i.e., QTL 12, 2B-1; QTL 39, 5B-3).

### Search for candidate genes

The search for candidate genes within the confidence interval of the major QTLs identified in the present study revealed some interesting correspondences (Table 3 and Figure S1). First of all, the *Ppd-B1* gene was found in the region of QTLs 10 and 11, as already suggested by its position in the genetic map. The wheat orthologous genes corresponding to *Constans4* (Griffiths et al., 2003), *Six-rowed spike 1* (*Vrs1*—Komatsuda et al., 2007), *CENTRORADIALS* (*CEN*—Comadran et al., 2012), and *SUSIBA2* (*SUGAR SIGNALLING IN BARLEY 2*—Su et al., 2015) were mapped within the interval of the QTL 12 on chromosome 2B, and their homoeologous copies were also mapped to chromosome 2A in correspondence of the QTL 6. Other correspondences with candidate genes were identified for QTLs of the group 3. The wheat gene *TaGID2* (Lou et al., 2016) and the wheat orthologous of the rice gene *HEXOKINASE9* (*HKX9*—Cho et al., 2006) were mapped to the same interval of the QTL 16 on chromosome 3A, while the wheat orthologous of the barley genes *FT2* (Faure et al., 2007), and *BRASSINOSTEROID-INSENSITIVE 1* (*BRI1*—Saisho et al., 2004), were located within the interval of the QTL 20 on chromosome 3B, together with the wheat gene *ENHANCED RESPONSE TO ABSCISIC ACID 1* (*ERA1*—Edae et al., 2013). The region of QTL 41 on chromosome 6A corresponded to the wheat gene *TaGw2* (Simmonds et al., 2014). Finally, two correspondences were also found for the group 7: the wheat orthologous of the rice gene *DWARF3* (Zhao et al., 2014) with the QTL 53 on chromosome 7A, and the barley one *Constans 6* (Griffiths et al., 2003) with the QTL 55 on chromosome 7B.

## Discussion

The physiological association between phenology and the yield components of tillering and spike fertility in wheat was analyzed at the molecular level using the QTLs already identified by Sanna et al. (2014), and several other QTLs identified in the same population through a novel field experiment and the use of an updated genetic map. Among these, the most interesting new QTLs for phenology were associated with the phyllochron in the SD-V and SD-FI (natural vernalization) plants. These QTLs were located on chromosomes 2B and 7A, and they explained a high proportion of the phenotypic variation for PHY (i.e., from 20 to 28 %). In particular, the *Ppd-B1* gene was found in the region of QTL 10 on linkage group 2B-1 (corresponding to QTL 4 of Sanna et al., 2014). This therefore confirms the key role of the variation in PHY in prolonging the sensitivity to the photoperiod of wheat plants after terminal spikelet formation (Miralles and Richards, 2000; Gonzáles et al., 2005; Sanna et al., 2014). In agreement with Sanna et al. (2014), who attributed the long TS-ANT of the SD-V plants to both the number of leaves emerging after TS and PHY, the two new QTLs for PHY defined in the present study were identified for the SD-grown plants.

### Tillering

Differences in tillering capacity (Lupton et al., 1974; Ali Dib et al., 1992) and in spike fertility (De Vita et al., 2007; Giunta et al., 2007) between old Italian cultivars, such as “Senatore Cappelli,” and modern Italian cultivars, such as “Ofanto,” gave origin to moderate to large genotypic variation in both the tillering and fertility traits, which depended on the flowering genes activated by the treatment applied.

Although tillering traits are indeed expected to be highly dependent on environmental factors (McMaster, 1997; Dreccer et al., 2013), the absence of nutrient and water limitations and the low plant density adopted here allowed us to demonstrate the genetic variability for tillering in durum wheat, as already seen for barley (Borràs et al., 2009; Alqudah et al., 2016) and bread wheat (Borràs-Gelonch et al., 2012). Both the phenotyping and the QTL analysis demonstrated a major role for the *Vrn* genes on the control of tillering, which was independent of any photoperiod or temperature effects. This was expected because the length of the vegetative period and the leaf number increase in plants when the vernalization requirements are not satisfied, which thus establishes more potential sites for tillering (Gomez-MacPherson and Richards, 1997). This was reflected in the higher MAXTILL and LN_MAXTILL_ of the non-vernalized plants, a trait that was associated with development *via* LN_TS_. The effect of the lack of vernalization on MAXTILL was much greater than that on FERTILL. Also, in the absence of resource limitations, the onset of stem elongation indeed establishes strong intra-plant competition, which results in senescence of the youngest tillers (McMaster, 1997). In any case, the RILs with more MAXTILL also showed higher FERTILL, as already discussed by Dreccer et al. (2013), and many QTLs controlling both traits were detected.

At the molecular level, the role of the *Vrn* genes on tillering was established by the co-location of the *Vrn-A1* locus with QTL 33 on chromosome 5A. This QTL was heavily involved in the control of tillering under the NV conditions, which confirms some previous studies (Snape et al., 1985; Roberts, 1989; Miura and Kuroshima, 1996; Kato et al., 2000; Kosner and Pankova, 2001; Cui et al., 2012; Jia et al., 2013; Xie et al., 2016), along with the meta-analysis carried out by Zhang et al. (2010). Consistent with our findings, Kato et al. (2000) identified three other QTLs along chromosome 5A that showed minor effects on tillering.

The LD-NV conditions also allowed the identification of significant QTLs for tillering traits on homology group 3 and linkage group 7A-1. In particular, QTL 20 on chromosome 3B is associated with MAXTILL and FERTILL, and represents a new, well-defined and specific QTL. Its importance for tillering was confirmed by the candidate gene analysis which mapped this QTL close to the wheat gene *ENHANCED RESPONSE TO ABSCISIC ACID 1* (*ERA1*). ERA1 was one of the five drought tolerance candidate genes studied by Edae et al. (2013) to identify functional markers involved in drought tolerance in bread wheat. In two environments *ERA1-B* was associated to spikes per square meter, and this association was detected under both dry and irrigated conditions. QTL 51 on chromosome 7A coincided with QTn.ipk-7A identified by Huang et al. (2004), with the common marker Xgwm276. It is interesting to note that QTL 51 was identified under the NV conditions, and Law (1996) localized the Vrn-B4 locus on chromosome 7. Moreover, the *Vrs1* gene mapped to chromosome 2A in correspondence of the QTL 6 confirms the role of this gene for tillering discussed in barley by Alqudah et al. (2016).

The only tillering trait where the variation was not associated with QTL 33 under the LD-NV conditions was RATE. A possible effect of the *Rht* genes on RATE emerged at the molecular level in the LD-V plants, which confirms the relationship between tillering and the *Rht* genes that was proposed by Irfaq et al. (2005); this demonstrated that semi-dwarf lines with *Rht-B1* or *Rht-D1* also show high productive tiller numbers. Under the SD-V conditions, RATE was also associated with QTL 31 on chromosome 5A, although this association explained only 11% of the phenotypic variation for this trait. This QTL is probably the same QTL as that reported previously by Jia et al. (2013) for tiller number (Qtn.nau-5A) on chromosome 5A, flanked by Xbarc56-Xgwm156.1.

In spite of the limiting photoperiod conditions of the SD-V environment, no QTL for tillering was associated with the region on chromosome 2B where the *Ppd* genes are located. This means that the chromosomal regions controlling the development via photoperiodism are different from those controlling tillering. The reason for this lack of effect of photoperiod sensitivity on tillering is that in this population the *Ppd* genes acted only on the length of the phenological phases subsequent to the tillering phase; i.e., terminal spikelet–anthesis (Sanna et al., 2014).

Many of the QTLs for tillering identified in this study that were not related with phenology and that each explained 18–19% of the phenotypic variance were located on chromosomes of homology groups 6 and 7, according to previous studies (Li et al., 2002; Huang et al., 2003; An et al., 2006; Kumar et al., 2007; Naruoka et al., 2011; Yang et al., 2013).

### Fertility

The impact on grain yield of variations in spikelet numbers induced by phasic development was shown by Rawson (1970), who demonstrated that grain yield per spike was dependent on spikelet numbers within each cultivar, when spikelet numbers were varied by daylength or vernalization conditions. The limiting daylength of the SD conditions had large positive effects on spikelet numbers, compared with the LD conditions, as was observed previously by Miralles and Richards (2000) and by Giunta et al. (2001). This appears to be due to the lengthening of the period available for spikelet initiation (Rawson, 1970; Arduini et al., 2009). The difference of about 10 spikelets per spike between the SD and LD conditions is comparable to the maximum difference in spikelet numbers between sowing dates observed by Arduini et al. (2009) in durum wheat sown at monthly intervals throughout a whole year. The large genotypic variation (i.e., range of 5.4 spikelets per spike) and relatively high heritability for spikelet number revealed by the limiting daylength of the SD environments was not associated with any developmental trait. In other words, changing the daylength produced an effect on SPKLT that was much larger than that seen when varying the genotypes within a daylength-limiting environment. However, this might be a consequence of the scarce genetic variability in the photoperiod sensitivity that was noted previously by Sanna et al. (2014) for this RIL population.

Genetic correlations highlighted a relationship between SPKLT and development only in the environment where *Eps* was driving development (i.e., the LD-V conditions). Also, the positive effects of high temperatures on SPKLT observed in the subsets can be interpreted as a result of *Eps*, because the actions of the *Eps* genes are modulated by temperature (Slafer and Rawson, 1995). Under the LD-V conditions, SPKLT was correlated with FLN and ANT, an association that was observed previously by Giunta et al. (2001) for both durum wheat and triticale. The *Eps* genes are believed to act via changes in the number of leaf or spikelet primordia initiated (Hoogendoorn, 1985) or the rate of primordia initiation (Gotoh, 1977), which thus establishes significant correlations between time to flowering and number of spikelets produced on the main stem (Worland et al., 1994; Worland, 1996). The data at the molecular level are consistent with this association, because the main QTLs associated with SPKLT overlapped with those associated with FLN and were mainly detected under the LD-V conditions on chromosomes 7A and 7B. One of these QTLs was common to QTL 14 previously identified by Sanna et al. (2014) for these same materials. Sanna et al. (2014) suggested a role for QTL 14 on *Eps*, a hypothesis that was also supported by Flood and Halloran (1983) and Hoogendoorn (1985), for which the *Eps* genes in bread wheat are localized on chromosome 7B. In the present study, QTL 55 on chromosome 7B was associated to FLN, and SPKLTms shared a common marker (wPt-8615) with QTL QKSk.ndsu.7B identified by Echeverry-Solarte et al. (2015), who associated this QTL with a series of yield-related traits and with the number of kernels per spikelet. An association between SPKLT and the *Eps* genes was also shown by Hu et al. (2015). Among a total of 22 significant associations for spike-related traits, Hu et al. (2015) associated one reliable SNP marker (BG314551_3_A_Y_162) with spikelet number on the main stem on chromosome arm 3AS in the same region as the *Eps* genes. Also, in diploid wheat, the chromosome region that includes the *Eps* locus *Eps-Am1* affects the duration of early developmental phases and the spikelet number (Lewis et al., 2008).

The *Vrn-A1* locus did not affect SPKLT in the present study, in contrast with Kato et al. (2000), who associated spikelet number with the *Vrn-A1* alleles on chromosome arm 5AL, and found two QTLs for spikelet number on the same arm. From a physiological point of view, cold requirements are not expected to be directly related to SPKLT, because spikelet primordia are produced once the cold requirements have been satisfied (Brown et al., 2013). On the other hand, the lower developmental rate caused by the lack of cold requirements satisfaction might indirectly change the number of spikelets initiated by moving the period of spikelet primordia induction to different photoperiod conditions.

K/SPKLT contributed greatly to the genetic variation in K/SPIKE in all of the environments, and was associated with *Rht-B1* on chromosome 4B. The most important consequence of the introduction of the *Rht* genes was the greater amount of dry matter allocated to the spikes at the expense of the stems (Austin et al., 1980; Chandler and Harding, 2013). Guo et al. (2017) demonstrated that in bread wheat this change allowed more spikelets to survive, and at the same time, it increased the survival of the distal florets within a spikelet; i.e., K/SPKLT. On the other hand, K/SPKLT was only weakly associated with phenology. Only under SD-FI conditions K/SPKLTms co-located with the phenological trait FLA on QTL 16, where the wheat orthologous of the rice gene *HEXOKINASE9* (*HXK9*—Cho et al., 2006), associated to spikelet fertility in Guo et al. (2017) was mapped. Environmental conditions around FLA can affect floret fertility (Fischer, 2011), particularly under field conditions, thus explaining the co-location of FLA and K/SPIKLT on QTL 16.

Variations in the other two components of spike fertility (i.e., CHAFF, K/CHAFF) were negatively associated at both the genetic and molecular levels. This is in contrast with Motzo et al. (2011), who showed no relationship between these two traits in triticale, but in agreement with Terrile et al. (2017) in bread wheat. The negative correlation between CHAFF and K/CHAFF reduces the possibility of exploiting the high heritability calculated for both traits in the combined ANOVA. Phenology had only limited effects on these two traits. The LD-V plants defined a positive relationship between CHAFF and SPKLT that was supported by the genetic correlations and by QTL 54, as indicated previously by Gaju et al. (2009) (indeed, more spikelets are expected to result in heavier spikes). QTL 54 was also indicative of an effect of phenology on CHAFF via FLA and ANT, which highlights the importance of the *Eps* genes for CHAFF.

Under the SD-V conditions, K/CHAFF was associated with ANT and FLN because the later anthesis arose in this environment through lengthening of the TS-ANT phase (Sanna et al., 2014); i.e., the period of spike growth. This is of key importance for spike fertility (Slafer et al., 2009).

The LD-NV plants suggested some effects of the *Vrn* genes on both CHAFF and K/CHAFF, because both of these traits co-located with most of the phenological traits on QTL 33. A role for the *Vrn* genes on CHAFF was also demonstrated by Kato et al. (2000), who showed significant effects of different regions of chromosome 5A on CHAFF. This apparent association can be interpreted as an indirect effect of the *Vrn* genes, as Sanna et al. (2014) demonstrated that they can extend their effects beyond terminal spikelet formation, in the TS-ANT phase, via an increase in the number of leaves emerging after TS, and consequently in PHY.

Following Guo and Schnurbusch (2015) the relationship between CHAFF and MAXTILL was explored. The two traits were negatively associated (*r* = −0.37, *P* < 0.001) only in the LD-NV environment, i.e., the environment with the highest MAXTILL and hence the greatest intra-plant competition.

As well as the pleiotropic effects that flowering genes can have on fertility traits, some of the QTLs identified in the present study were not related to development. For K/SPIKEms, QTL 26 on chromosome 4A that was associated with this trait coincided with QKS.ndsu.4A defined by Echeverry-Solarte et al. (2015), as demonstrated by the common marker wPt-2247. Moreover, QTL 14 on chromosome 2B-3 associated to K/SPIKEms and K/SPIKEtill was only 5 cM from SNP IAAV1502 that was associated with tiller number in the study of Guo et al. (2017). The association between K/SPIKE and the QTL 55, where the *CONSTANS6* gene (Griffiths et al., 2003) was located, confirms the association found by Guo et al. (2017) between *CONSTANS6*, floret primordia loss and maximum floret number. Also, QTL 21 (chromosome 3B-1) was associated with K/SPKLTms and was located about 20 cM from SNP IWB11679 identified by Guo et al. (2017) for grain number, grains per spike, and tiller and grain number per spikelet. The results of the association mapping for CHAFF also appeared particularly useful. Indeed, in the present study in addition to known regions on chromosome 5A (Guo et al., 2017), we identified new genetic regions on chromosomes 7A (QTL 53), 7B (QTL 54), and 6A (QTL 41) with strong PVE (from 17 to 22%). The mapping of *Vrs1* close to QTL 12, which explains 18% of the phenotypic variation in K/CHAFF, might depend on the role of *Vrs1* protein, which suppresses floret primordia development within wheat spikelets thus influencing grain number.

## Conclusions

Phenotyping of both phenology and kernel number according to a robust physiological model amplified the possibility to identify the genetic factors underlying their variations, and at the same time facilitated the analysis of how phenology impacts on kernel number at the genetic level. Moreover, isolation of known flowering gene cues by manipulation of the environmental conditions provided the opportunity for each group of genes to be expressed without confounding effects of the others, and for the definition of the interactions of each QTL with the environment. This was particularly useful for the *Eps* genes, which usually have smaller effects than those associated with sensitivity to either photoperiod or vernalization. Unfortunately, the low level of photoperiod sensitivity that characterized the specific RIL population analyzed limited the possibility to detect pleiotropic effects of the *Ppd* genes on kernel number.

Additional studies on allelic variations of developmental genes using wheat germplasm collections will generalize to a great extent our knowledge of their effects on the yield-related traits of durum wheat.

## Author contributions

FG and RM: Conceived the project, designed the field and pot experiments, analyzed the pertaining data and wrote the article with contributions of all authors; GS: Supervised the field and pot experiment and acquired field and pot data; AM and PD: Provided the recombinant inbred line population, performed the genetic analysis, and complemented the writing on the genetic topics.

### Conflict of interest statement

The authors declare that the research was conducted in the absence of any commercial or financial relationships that could be construed as a potential conflict of interest.

## Abbreviations

ANT: time of anthesis
CHAFF: chaff weight
FERTTILL: number of fertile tillers
FERTTILL%: percentage of fertile tillers
FLN: final leaf number
FLA: flag leaf appearance
K/CHAFF: number of kernels per g chaff
K/SPIKE: number of kernels per spike
K/SPKLT: number of kernels per fertile spikelet
LN_MAXTILL_: leaf number when MAXTILL reached
MAXTILL: maximum number of tillers
PHY: phyllochron
PVE: phenotypic variation explained
QTLs: quantitative trait loci
RATE = MAXTILL/LN_MAXTILL_: rate of tillering
RIL: recombinant inbred line
SPKLT: number of total spikelets
TS: terminal spikelet stage.
