# Supplementary material for: Environmental and Genetic Variation for Yield-Related Traits of Durum Wheat as Affected by Development
Source: Front Plant Sci. 2018 Jan 18;9:8. doi: 10.3389/fpls.2018.00008 (PMC5778143; doi:10.3389/fpls.2018.00008)
Supplement: Supplementary file 1 [file Table1.DOCX]

**Table S1.** ANOVA and heritability of the various tillering traits expressed in the ‘Ofanto’ × ‘Senatore Cappelli’ RIL population.

| Trait | LD-V | | | | | | | LD-NV | | | | | | | SD-V | | | | | | |
| --- | --- | --- | --- | --- | --- | --- | --- | --- | --- | --- | --- | --- | --- | --- | --- | --- | --- | --- | --- | --- | --- |
|  | Genotype | | | Residual | | | *h^2^* | Genotype | | | Residual | | | *h*^2^ | Genotype | | | Residual | | | *h*^2^ |
| MAXTILL (n) | 0.499 | ± | 0.123 | 1.72 | ± | 0.12 | ***59.4*** | 4.96 | ± | 1.055 | 5.86 | ± | 0.6 | ***71.1*** | 0.658 | ± | 0.14 | 1.12 | ± | 0.09 | ***70.3*** |
| FERTILL (n) | 0.077 | ± | 0.028 | 0.56 | ± | 0.04 | ***40.8*** | 0.708 | ± | 0.165 | 1.06 | ± | 0.11 | ***65.5*** | 0.639 | ± | 0.13 | 0.86 | ± | 0.07 | ***75.0*** |
| FERTILL (%) | 66.4 | ± | 18.3 | 286 | ± | 20.4 | ***53.5*** | 25.21 | ± | 7.32 | 63.7 | ± | 6.57 | ***52.9*** | 25.6 | ± | 15.6 | 321 | ± | 25.7 | ***24.4*** |
| HS_MAXTILL_ | 0.135 | ± | 0.08 | 1.92 | ± | 0.14 | ***25.5*** | 0.385 | ± | 0.228 | 3.02 | ± | 0.31 | ***26.2*** | 2.176 | ± | 0.5 | 4.63 | ± | 0.38 | ***65.5*** |
| RATE (tillers leaf^-1^) | 0.007 | ± | 0.00 | 0.03 | ± | 0.00 | ***57.0*** | 0.024 | ± | 0.008 | 0.07 | ± | 0.01 | ***50.9*** | 0.002 | ± | 0.001 | 0.01 | ± | 0.001 | ***46.3*** |
|  | Combined analysis | | | | | | | | | |  |  |  |  |  |  |  |  |  |  |  |
|  | Genotype | | | GxE | | |  | Residual | | | *h*^2^ |  |  |  |  |  |  |  |  |  |  |
| MAXTILL (no) | 0 | ± | 0 | 2.11 | ± | 0.29 |  | 2.439 | ± | 0.115 | ***0.0*** |  |  |  |  |  |  |  |  |  |  |
| FERTILL (no) | 0.002 | ± | 0.041 | 0.45 | ± | 0.07 |  | 0.81 | ± | 0.038 | ***0.5*** |  |  |  |  |  |  |  |  |  |  |
| FERTILL (%) | 3.8 | ± | 6.5 | 34.5 | ± | 10.5 |  | 252.8 | ± | 11.9 | ***4.8*** |  |  |  |  |  |  |  |  |  |  |
| HS_MAXTILL_ | 0.102 | ± | 0.111 | 0.78 | ± | 0.17 |  | 3.141 | ± | 0.149 | ***8.7*** |  |  |  |  |  |  |  |  |  |  |
| RATE (tillers leaf^-1^) | 0 | ± | 0 | 0.01 | ± | 0.002 |  | 0.03 | ± | 0.001 | ***0.0*** |  |  |  |  |  |  |  |  |  |  |

The analysis was performed both within each treatment separately, and also on the combined data-set.
