# Supplementary material for: Environmental and Genetic Variation for Yield-Related Traits of Durum Wheat as Affected by Development
Source: Front Plant Sci. 2018 Jan 18;9:8. doi: 10.3389/fpls.2018.00008 (PMC5778143; doi:10.3389/fpls.2018.00008)
Supplement: Supplementary file 2 [file Table2.DOCX]

**Table S2.** ANOVA for heritability of the various fertility traits expressed in the ‘Ofanto’ × ‘Senatore Cappelli’ RIL population.

| **Trait** | **LD-V** | | | **LD-NV** | | | | **SD-V** | | | **SD-FI** | | |
| --- | --- | --- | --- | --- | --- | --- | --- | --- | --- | --- | --- | --- | --- |
|  | **Genotype** | **Residual** | **h^2^** | **Genotype** | **Residual** | | **h^2^** | **Genotype** | **Residual** | **h^2^** | **Genotype** | **Residual** | **h^2^** |
| **Main stem** | | | | | | | | | | | | | |
| SPKLT | 0.733 ±0.147 | 1.435 ±0.1 | **72.3** | 0.58 ±0.17 | 1.493 ±0.153 | | **52.6** | 1.429 ±0.267 | 1.635 ±0.13 | **79.1** | 0.885 ±0.266 | 4.477 ±0.32 | **49.3** |
| K/SPIKE | 9.81 ±2.75 | 45.59 ±3.19 | **52.4** | 39.64 ±8.81 | 50.81 ±5.31 | | **68.8** | 45.78 ±9.05 | 64.49 ±5.02 | **75.3** | 19.4 ±6.6 | 121.4 ±8.70 | **44.0** |
| K/SPKLT | 0.0422 ±0.012 | 0.203 ±0.0142 | **51.6** | 0.1183 ±0.0293 | 0.203 ±0.0212 | | **62.2** | 0.04866 ±0.01 | 0.081 ±0.01 | **72.2** | 0.0233 ±0.0099 | 0.211 ±0.02 | **35.2** |
| CHAFF | 0.00609 ±0.002 | 0.0225 ±0.002 | **58.1** | 0.00852 ±0.002 | 0.014 ±0.001 | | **94.7** | 0.02786 ±0.0058 | 0.049 ±0.00 | **71.1** | 0.0243 ±0.0065 | 0.096 ±0.01 | **55.5** |
| K/CHAFF | 49.8 ±11.2 | 139.4 ±9.7 | **64.7** | 127.5 ±26.5 | 134.2 ±13.9 | | **73.0** | 76.93 ±13.6 | 63.18 ±4.96 | **83.8** | 34.62 ±6.83 | 60.75 ±4.36 | **73.7** |
| **Tillers** | | | | | | | | | | |  |  |  |
| K/SPIKE | 1.18 ±1.67 | 42.93 ±3.28 | **10.8** | 7.78 ±3.1 | 31.68 ±3.45 | | **39.2** | 22.91 ±5.49 | 60.57 ±4.67 | **62.2** |  |  |  |
| CHAFF | 0.00387 ±0.0012 | 0.0174 ±0.0014 | **49.2** | 0.00153 ±0.00116 | 0.015 ±0.0017 | | **20.6** | 0.01222 ±0.0029 | 0.031 ±0.00 | **62.6** |  |  |  |
| K/CHAFF | 37.9 ±11.3 | 166.5 ±12.9 | **49.8** | 98.2 ±23.4 | 145.2 ±15.8 | | **64.3** | 75.28 ±13.81 | 75.88 ±5.95 | **80.9** |  |  |  |
|  | **Combined analysis** | | | | |  |  |  |  |  |  |  |  |
|  | **Genotype** | **G × E** |  | **Residual** | **h^2^** |  |  |  |  |  |  |  |  |
| **Main stem** | | | | | |  |  |  |  |  |  |  |  |
| SPKLT | 0.191 ±0.078 | 0.678 ±0.107 |  | 2.395 ±0.093 | **20.8** |  |  |  |  |  |  |  |  |
| K/SPIKE | 0.50 ±1.41 | 22.08 ±2.67 |  | 38.43 ±1.5 | **3.3** |  |  |  |  |  |  |  |  |
| K/SPKLT | 0.09 ±0.58 | 4.97 ±1.14 |  | 35.27 ±1.36 | **0.9** |  |  |  |  |  |  |  |  |
| CHAFF | 0.004 ±0.0016 | 0.0117 ±0.002 |  | 0.0501±0.0019 | **22.9** |  |  |  |  |  |  |  |  |
| K/CHAFF | 14.23 ±5.19 | 54.34 ±6.63 |  | 97.03 ±3.78 | **28.2** |  |  |  |  |  |  |  |  |
| **Tillers** | | | | | |  |  |  |  |  |  |  |  |
| K/SPIKE | 0.95 ±1.53 | 10.15 ±2.45 |  | 48.14 ±2.3 | **5.6** |  |  |  |  |  |  |  |  |
| CHAFF | 0.002 ±0.0009 | 0.004 ±0.001 |  | 0.0228 ±0.0 | **23.1** |  |  |  |  |  |  |  |  |
| K/CHAFF | 268.1 | 43.1 ±8.3 |  | 126.8 ±6.2 | **35.3** |  |  |  |  |  |  |  |  |

The analysis was performed both within each treatment separately, and also on the combined data-set.
