# Supplementary material for: Environmental and Genetic Variation for Yield-Related Traits of Durum Wheat as Affected by Development
Source: Front Plant Sci. 2018 Jan 18;9:8. doi: 10.3389/fpls.2018.00008 (PMC5778143; doi:10.3389/fpls.2018.00008)
Supplement: Supplementary file 3 [file Image1.pdf]

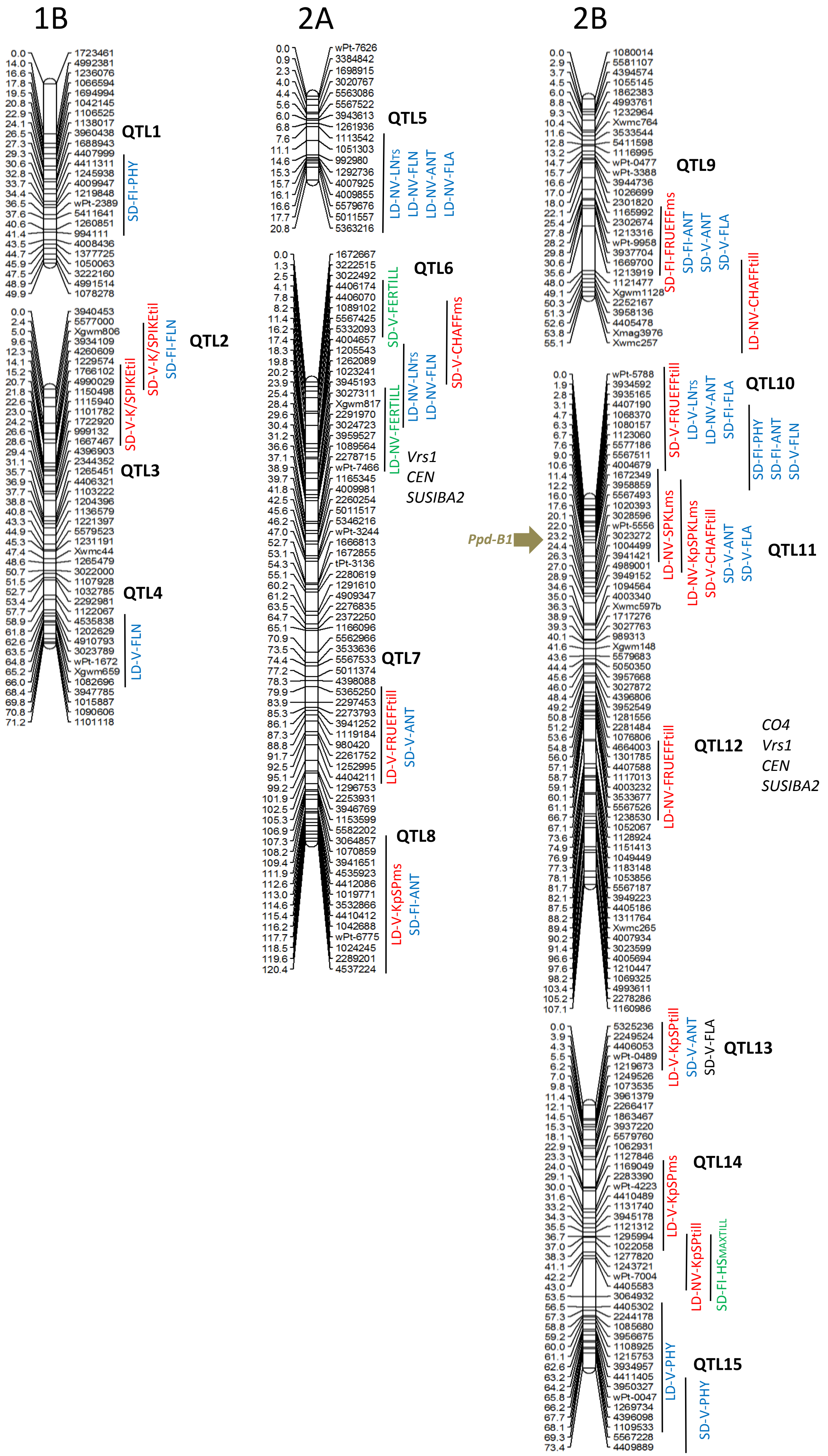

1B
2A
2B
QTL1
SD-FI-PHY
QTL2
SD-V-K/SPIKEtil
SD-FI-FLN
SD-V-K/SPIKEtil
QTL3
QTL4
LD-V-FLN
QTL5
LD-NV-LNTS
LD-NV-FLN
LD-NV-ANT
LD-NV-FLA
QTL6
SD-V-FERTILL
LD-NV-LNTS
LD-NV-FLN
SD-V-CHAFFms
LD-NV-FERTILL
Vrs1
CEN
SUSIBA2
QTL7
LD-V-FRUEFFtill
SD-V-ANT
QTL8
LD-V-KpSPms
SD-FI-ANT
QTL9
SD-FI-FRUEFFms
SD-FI-ANT
SD-V-ANT
SD-V-FLA
LD-NV-CHAFFtill
QTL10
SD-V-FRUEFFtill
LD-V-LNTS
LD-NV-ANT
SD-FI-FLA
SD-FI-PHY
SD-FI-ANT
SD-V-FLN
Ppd-B1
QTL11
LD-NV-SPKLms
LD-NV-KpSPKLms
SD-V-CHAFFtill
SD-V-ANT
SD-V-FLA
QTL12
LD-NV-FRUEFFtill
CO4
Vrs1
CEN
SUSIBA2
QTL13
LD-V-KpSPtill
SD-V-ANT
SD-V-FLA
QTL14
LD-V-KpSPms
LD-NV-KpSPtill
SD-FI-HSMAXTILL
QTL15
LD-V-PHY
SD-V-PHY

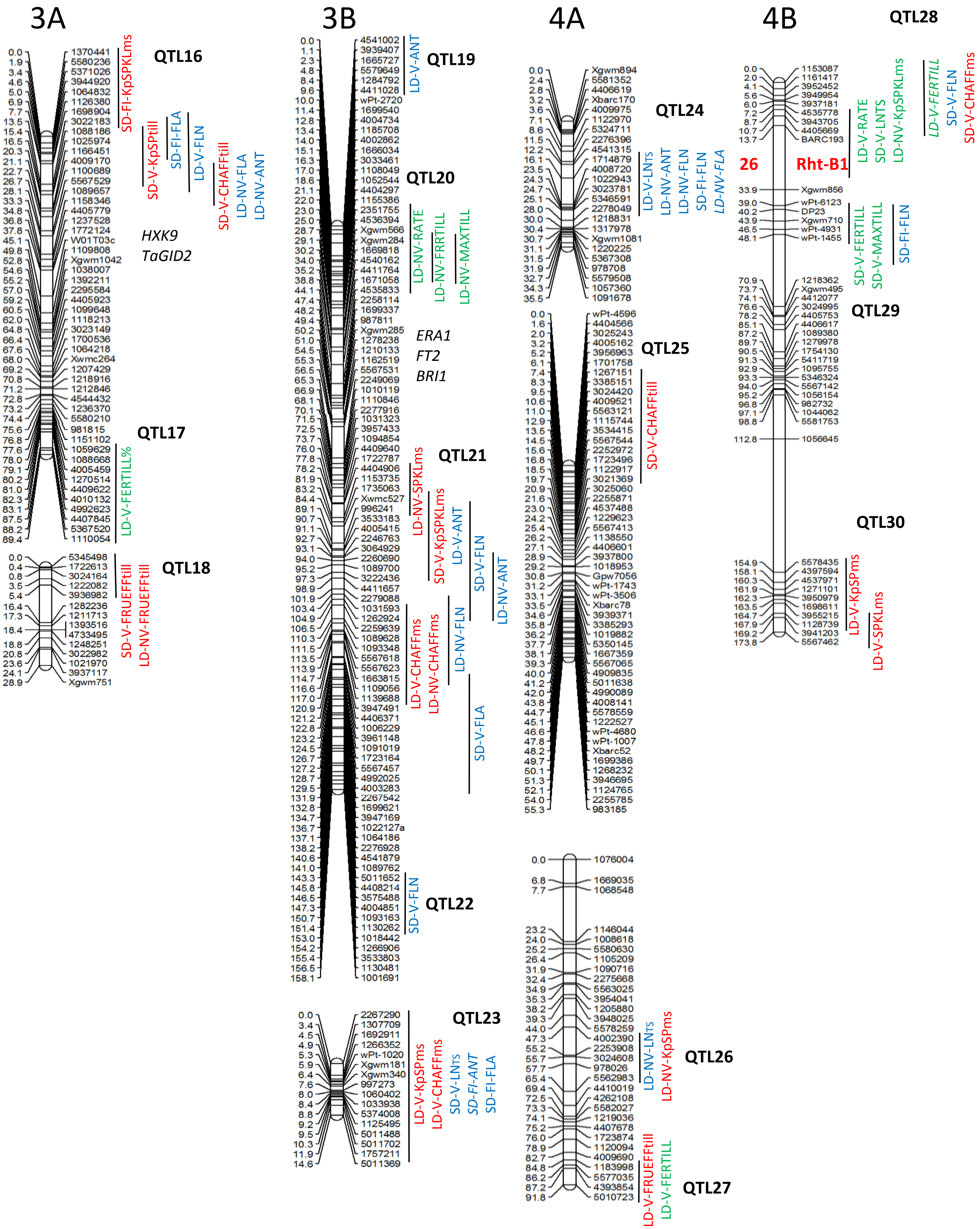

3A
QTL16
SD-FI-KpSPKLms
SD-V-KpSPtill
SD-FI-FLA
SD-V-FLN
SD-V-CHAFFtill
LD-NV-FLA
LD-NV-ANT
HXK9
TaGID2
QTL17
LD-V-FERTILL%
QTL18
SD-V-FRUEFFtill
LD-NV-FRUEFFtill
3B
QTL19
LD-V-ANT
QTL20
LD-NV-RATE
LD-NV-FRRTILL
LD-NV-MAXTILL
ERA1
FT2
BRI1
QTL21
LD-NV-SPKLms
SD-V-KpSPKLms
LD-V-ANT
SD-V-FLN
SD-NV-ANT
LD-NV-FLN
LD-V-CHAFFms
LD-NV-CHAFFms
SD-V-FLA
QTL22
SD-V-FLN
QTL23
LD-V-KpSPms
LD-V-CHAFFms
SD-V-LNTS
SD-FI-ANT
SD-FI-FLA
4A
QTL24
LD-V-LNTS
LD-NV-ANT
LD-NV-FLN
SD-FI-FLN
LD-NV-FLA
QTL25
SD-V-CHAFFtill
QTL26
LD-NV-LNTS
LD-NV-KpSPms
QTL27
LD-V-FRUEFFtill
LD-V-FERTILL
4B
QTL28
26
Rht-B1
LD-V-RATE
SD-V-LNTS
LD-NV-KpSPKLms
LD-V-FERTILL
SD-V-FLN
SD-V-CHAFFms
SD-V-FERTILL
SD-V-MAXTILL
SD-FI-FLN
QTL29
QTL30
LD-V-KpSPms
LD-V-SPKLms

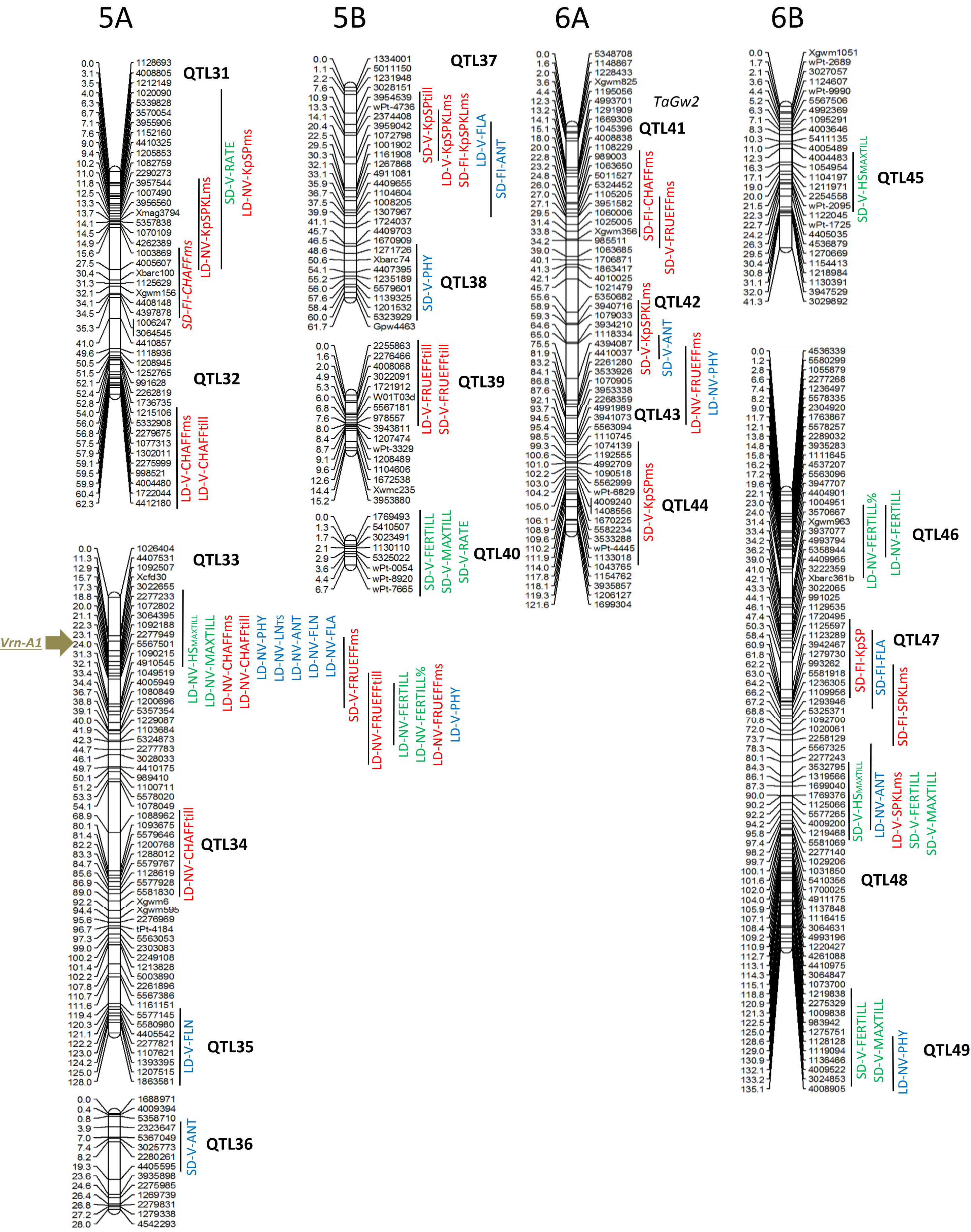
5A
5B
6A
6B
QTL31
SD-V-RATE
LD-NV-KpSPms
LD-NV-KpSPKLms
SD-FI-CHAFFms
QTL32
LD-V-CHAFFms
LD-V-CHAFFtill
QTL33
Vrn-A1
LD-NV-HSMAXTILL
LD-NV-MAXTILL
LD-NV-CHAFFms
LD-NV-CHAFFtill
LD-NV-PHY
LD-NV-LNTS
LD-NV-ANT
LD-NV-FLN
LD-NV-FLA
SD-V-FRUEFFms
LD-NV-FRUEFFtill
LD-NV-FERTILL
LD-NV-FERTILL%
LD-NV-FRUEFFms
LD-V-PHY
QTL34
LD-NV-CHAFFtill
QTL35
LD-V-FLN
QTL36
SD-V-ANT
QTL37
SD-V-KpSPtill
LD-V-KpSPKLms
SD-FI-KpSPKLms
LD-V-FLA
SD-FI-ANT
QTL38
SD-V-PHY
QTL39
LD-V-FRUEFFtill
SD-V-FRUEFFtill
QTL40
SD-V-FERTILL
SD-V-MAXTILL
SD-V-RATE
TaGw2
QTL41
SD-FI-CHAFFms
SD-V-FRUEFFms
QTL42
SD-V-KpSPKLms
SD-V-ANT
LD-NV-FRUEFFms
LD-NV-PHY
QTL43
QTL44
SD-V-KpSPms
QTL45
SD-V-HSMAXTILL
QTL46
LD-NV-FERTILL%
LD-NV-FERTILL
QTL47
SD-FI-KpSP
SD-FI-FLA
SD-FI-SPKLms
QTL48
SD-V-HSMAXTILL
LD-NV-ANT
LD-V-SPKLms
SD-V-FERTILL
SD-V-MAXTILL
QTL49
SD-V-FERTILL
SD-V-MAXTILL
LD-NV-PHY

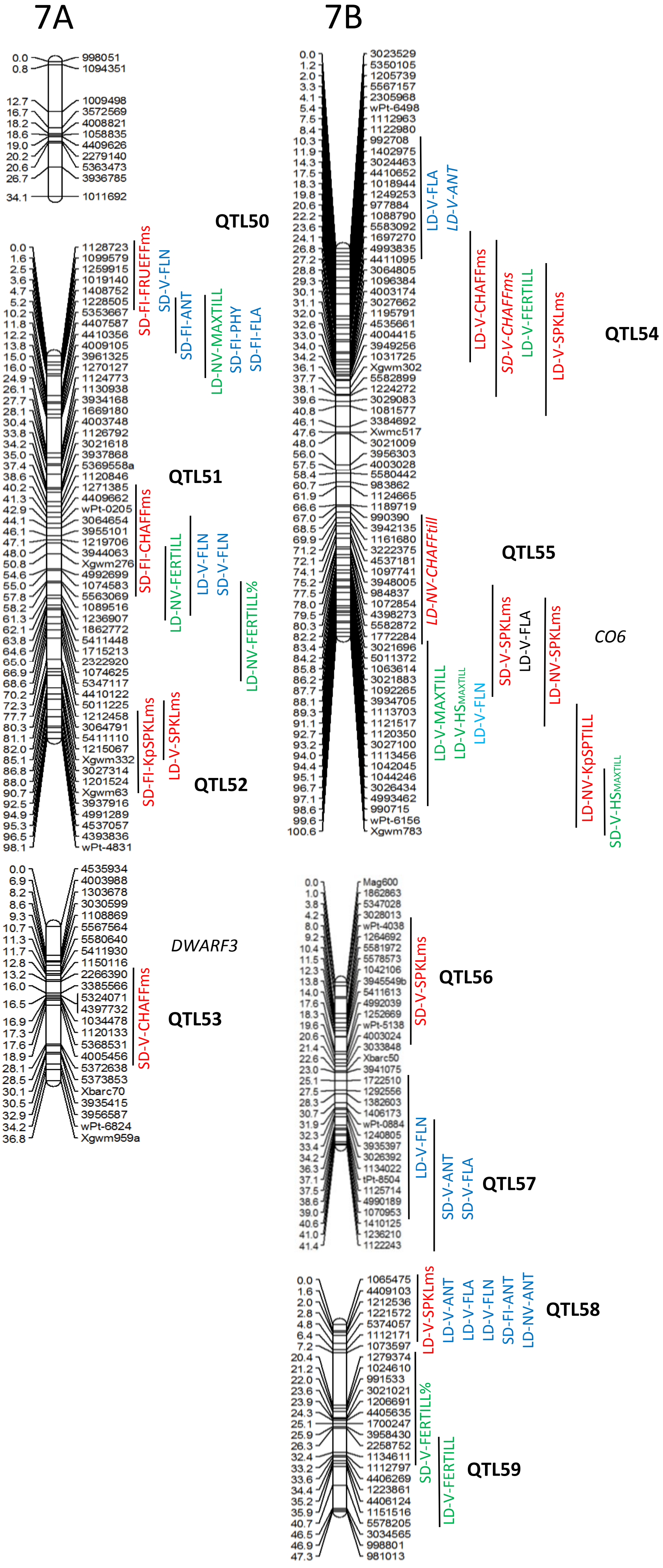

7A
7B
QTL50
SD-FI-FRUEFFms
SD-V-FLN
SD-FI-ANT
LD-NV-MAXTILL
SD-FI-PHY
SD-FI-FLA
QTL51
SD-FI-CHAFFms
LD-NV-FERTILL
LD-V-FLN
SD-V-FLN
LD-NV-FERTILL%
SD-FI-KpSPKLms
LD-V-SPKLms
QTL52
DWARF3
SD-V-CHAFFms
QTL53
LD-V-FLA
LD-V-ANT
LD-V-CHAFFms
SD-V-CHAFFms
LD-V-FERTILL
LD-V-SPKLms
QTL54
LD-NV-CHAFFtill
QTL55
SD-V-SPKLms
LD-V-FLA
LD-NV-SPKLms
CO6
LD-V-MAXTILL
LD-V-HSMAXTILL
LD-V-FLN
LD-NV-KpSPTILL
SD-V-HSMAXTILL
SD-V-SPKLms
QTL56
LD-V-FLN
SD-V-ANT
SD-V-FLA
QTL57
LD-V-SPKLms
LD-V-ANT
LD-V-FLA
LD-V-FLN
SD-FI-ANT
LD-NV-ANT
QTL58
SD-V-FERTILL%
LD-V-FERTILL
QTL59

Figure S1. Chromosomal regions harbouring QTLs for phenological (blue), tillering (green) and fertility (red) traits. The suffix ‘ms’ stands for main stem, ‘till’ for tiller.
